# Linking EORTC QLQ-C-30 and PedsQL/PEDQOL physical functioning scores in patients with osteosarcoma^☆^

**DOI:** 10.1016/j.ejca.2022.03.018

**Published:** 2022-07

**Authors:** Axel Budde, Katja Baust, Leonie Weinhold, Mark Bernstein, Stefan Bielack, Catharina Dhooge, Lars Hjorth, Katherine A. Janeway, Meriel Jenney, Mark D. Krailo, Neyssa Marina, Rajaram Nagarajan, Sigbjørn Smeland, Matthew R. Sydes, Patricia De Vos, Jeremy Whelan, Andreas Wiener, Gabriele Calaminus, Matthias Schmid

**Affiliations:** a Department of Paediatric Haematology and Oncology, University Hospital Bonn, Bonn, Germany; b Department of Medical Biometry, Informatics, and Epidemiology, University Hospital Bonn, Bonn, Germany; c IWK Health Centre, Dalhousie University, Halifax, NS, Canada; d Zentrum für Kinder-, Jugend- und Frauenmedizin, Pädiatrie, Klinikum Stuttgart, Olgahospital, Stuttgart, Germany; e Department of Internal Medicine and Paediatrics, Faculty of Medicine and Health Sciences, Ghent University Hospital, Ghent, Belgium; f Department of Clinical Sciences, Department of Paediatrics, Lund University, Skane University Hospital, Lund, Sweden; g Dana-Farber/Boston Children's Cancer and Blood Disorders Center, Harvard Medical School, Boston, MA, USA; h Women's Services Clinical Board, University Hospital of Wales, Cardiff, UK; i Statistics and Data Center, Children's Oncology Group, Monrovia, CA, USA; j Five Prime Therapeutics, South San Francisco, CA, USA; k Division of Oncology, Cincinnati Children's Hospital Medical Center, University of Cincinnati College of Medicine, Cincinnati, OH, USA; l Norwegian Radium Hospital, Oslo University Hospital, Oslo, Norway; m MRC Clinical Trials Unit at UCL, Institute of Clinical Trials and Methodology, University College London, London, UK; n Department of Paediatric Haematology and Oncology, Ghent University Hospital, Ghent, Belgium; o Department of Oncology, University College Hospital, London, UK; p West German Proton Therapy Center Essen, Essen, Germany

**Keywords:** Childhood cancer, EORTC QLQ-C30, Patient-reported outcome (PRO), Paediatric quality of life inventory (PedsQL), Paediatric quality of life questionnaire (PEDQOL), Physical functioning quality-of-life (QoL), Score linking, COG, Children's Oncology Group, COSS, Cooperative Osteosarcoma Group, EOI, European Osteosarcoma Intergroup, EORTC QLQ-C30, European Organisation for Research and Treatment of Cancer Core Questionnaire, EURAMOS-1, EUropean AMerican Osteosarcoma Study-1, FACT-G, Functional Assessment of Cancer Therapy - General, IRT, Item Response Theory, LOA, Limit of Agreement, PEDQOL, Paediatric Quality Of Life Questionnaire, PedsQL, Paediatric Quality of Life Inventory, PRO, Patient-Reported Outcome, PROMIS, Patient-Reported Outcomes Measurement Information System, QoL, Quality-of-Life, SSG, Scandinavian Sarcoma Group

## Abstract

**Purpose:**

The available questionnaires for quality-of-life (QoL) assessments are age-group specific, limiting comparability and impeding longitudinal analyses. The comparability of measurements, however, is a necessary condition for gaining scientific evidence. To overcome this problem, we assessed the viability of harmonising data from paediatric and adult patient-reported outcome (PRO) measures.

**Method:**

To this end, we linked physical functioning scores from the Paediatric Quality of Life Inventory (PedsQL) and the Paediatric Quality of Life Questionnaire (PEDQOL) to the European Organisation for Research and Treatment of Cancer Core Questionnaire (EORTC QLQ-C30) for adults. Samples from the EURAMOS-1 QoL sub-study of 75 (PedsQL) and 112 (PEDQOL) adolescent osteosarcoma patients were concurrently administered both paediatric and adult questionnaires on 98 (PedsQL) and 156 (PEDQOL) occasions. We identified corresponding scores using the single-group equipercentile linking method.

**Results:**

Linked *physical functioning* scores showed sufficient concordance to the EORTC QLQ-C30: Lin's *ρ* = 0.74 (PedsQL) and Lin's *ρ* = 0.64 (PEDQOL).

**Conclusion:**

Score linking provides clinicians and researchers with a common metric for assessing QoL with PRO measures across the entire lifespan of patients.

## Highlights

• Score linking is a promising method for linking physical functioning scores obtained with paediatric and adult quality-of-life (QoL) questionnaires in a population of survivors of childhood osteosarcoma.
• The method provides methodological rigour for quality-of-life (QoL) assessments across the lifespan of cancer patients when the use of distinct age-group-specific inventories is unavoidable.
• The approach may create the conditions for conducting longitudinal mixed-model meta-analyses.

## 1 Introduction

Quality-of-life (QoL) data are generally collected by self-report questionnaires. Health-related QoL questionnaires can be age-group specific. This age group specificity limits comparability and impedes numerical longitudinal analysis, especially if different instruments are needed to span the age range of the study. Specifically, the motivation for linking scores from paediatric and adult instruments was to make them comparable on a common scale, allowing the study of the QoL developmental trajectory continuously and permitting the analysis with mixed models.

The use of different instruments constitutes a considerable hurdle for the analysis and interpretation of QoL data, since “[t]he comparability of measurements made in differing circumstances by different methods and investigators is a fundamental pre-condition for all of science” [1]. Therefore, valid methods for linking scores are required.

Dorans provides an overview of applying linking methodology within the realm of patient-reported outcome (PRO) measures [2] (Table 1a).

**Table 1:** Publications on linking PRO measures.

Publications
**Adults**
Health status [[3], [4], [5], [6]]
Physical functioning [7, 8]
Physical and mental health summary scores [9]
Self-regulation [10]
Depression [[11], [12], [13], [14], [15]]
Pain [16]
Pain interference [17]
Anxiety [[18], [19], [20], [21]]
Fatigue [20, 21]
EORTC QLQ-C30 <> FACT-G [22]
**Children <> Adults**
Emotional distress [23]
Physical functioning in a population of individuals with spinal cord injury [24]

In the present study, we evaluated the viability of linking *physical functioning* scores of two paediatric PRO questionnaires (the PedsQL and the PEDQOL) to the EORTC QLQ-C30) in a population of survivors of childhood osteosarcoma. We restrict our report to the *physical functioning* domain because we were mainly interested in the viability of linking paediatric and adult instruments. We provide information on linking *emotional functioning*, *cognitive functioning*, *social functioning*, *fatigue* and *pain* domains in the appendix.

## 2 Materials and methods

The overall study design [25,26] and the methodological specifics of the QoL questionnaire sub-study have been laid out in detail previously [27]. We briefly describe the study design.

### 2.1 Participants

**Fig. 1:**
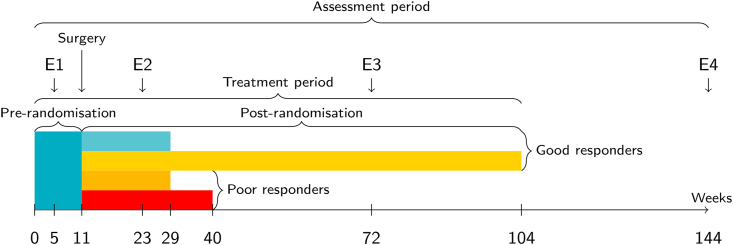
**Timeline for QoL assessments**.

The EURAMOS-1 trial cohort consisted of 2260 participants who, between the ages 5 and 40 years old, had been diagnosed with a previously untreated resectable high-grade osteosarcoma (at any site, except for craniofacial structures). Among these, 2213 participants were eligible for QoL-assessment (≥5 years old) and had a questionnaire in their respective language available (see [27]). Recruitment took place between 2005 and 2011, involving 17 countries and four study groups: the Children's Oncology Group (COG), the Cooperative Osteosarcoma Group (COSS), the European Osteosarcoma Intergroup (EOI), and the Scandinavian Sarcoma Group (SSG). EURAMOS-1 consortium members and their affiliations are listed in Appendix E.1. We obtained demographics from the EURAMOS-1 enrolment survey (sex, date of birth, and study group). Age was stratified as “5 to 15”, “16 to 17” and “18 or older”. As a secondary outcome measure, QoL was assessed prospectively at four time points during and after treatment (Fig. 1).

### 2.2 Questionnaires

Due to the unavailability of a single questionnaire suited for use across the whole age span of participants and in all participating countries, the EURAMOS-1 consortium opted for using different, age- and country-specific instruments (Table 2a).

**Table 2a:** QoL questionnaire by region and age group.

Questionnaire	Region	Age group		
		≥5 −15	16–17	≥18
PedsQL	COG (North America) & EOI (North West Europe)	+	+	+
PEDQOL	COSS (Central Europe) & SSG (Scandinavia)	+	+	+
EORTC QLQ-C30	All	+	+	+

In the age range 16–18 years old, all patients were asked to complete a paediatric questionnaire (either PedsQL [28] or PEDQOL [29]) and the EORTC QLQ-C30 [30]. We used this sub-sample for score linking. We restricted our study to aggregate scores pertaining to physical functioning, given its significance to QoL in osteosarcoma survivors and the substantial conceptual overlap between instruments in this domain. We linked two sub-sets of participants aged 16–17 years. These sub-sets were administered either the PedsQL or the PEDQOL questionnaire before the EORTC QLQ-C30 on the same day.

### 2.3 Analyses

#### 2.3.1 Similarity of item content and physical functioning sub-scale structure between instruments

The PedsQL, the PEDQOL and the EORTC QLQ-C30 all contain items that assess the physical functioning domain with multiple items (for details on scoring, see Table 2b and for verbatim item content see Appendix F).

**Table 2b:** QoL questionnaires physical functioning scoring.

Questionnaire	Number of items	Scale points	Period
PedsQL	4	5	Past month
PEDQOL	4	4	Past week
EORTC QLQ-C30	5	4	Past week

Item content showed substantial overlap across the three measures. To measure internal consistency of the instruments, we calculated Cronbach’s α. A summary of the results is given in Table 2c.

**Table 2c:** Internal consistency reliability of the physical functioning aggregate scores of the three instruments.

Time point	Questionnaire	Linked to	N^1^	Cronbach's α (95% CI)	Item–total correlation^2^		
					Min	Mean	Max
E1	PedsQL	EORTC QLQ-C30	38	0.87 (0.80, 0.93)	0.43	0.69	0.82
	PEDQOL		41	0.68 (0.52, 0.85)	0.49	0.60	0.68
	EORTC QLQ-C30	PedsQL	38	0.80 (0.70, 0.89)	0.35	0.65	0.79
		PEDQOL	41	0.88 (0.82, 0.94)	0.62	0.77	0.86
E2	PedsQL	EORTC QLQ-C30	24	0.73 (0.57, 0.88)	0.28	0.51	0.82
	PEDQOL		47	0.47 (0.22, 0.72)	0.06	0.48	0.68
	EORTC QLQ-C30	PedsQL	24	0.73 (0.58, 0.87)	0.26	0.61	0.75
		PEDQOL	47	0.82 (0.73, 0.90)	0.53	0.68	0.77
E3	PedsQL	EORTC QLQ-C30	20	0.77 (0.63, 0.92)	0.42	0.57	0.67
	PEDQOL		41	0.60 (0.40, 0.80)	0.42	0.54	0.59
	EORTC QLQ-C30	PedsQL	20	0.76 (0.61, 0.91)	0.43	0.70	0.85
		PEDQOL	41	0.78 (0.69, 0.87)	0.45	0.67	0.80
E4	PedsQL	EORTC QLQ-C30	16	0.86 (0.76, 0.95)	0.34	0.68	0.88
	PEDQOL		27	0.65 (0.44, 0.86)	0.28	0.59	0.74
	EORTC QLQ-C30	PedsQL	16	0.85 (0.74, 0.95)	0.60	0.79	0.90
		PEDQOL	27	0.74 (0.59, 0.89)	0.31	0.64	0.75

^1^ N refers to the number of participants in which Cronbach's α was measured for the instrument in the second column when linked to the instrument in the third column.

^2^ Item–total correlation indicates the correlation between the score on a single item and the aggregate physical functioning sub-scale score.

#### 2.3.2 Summary of physical functioning raw scores, correlation and concordance between instruments

The overall mean physical functioning score, i.e. across all four time points, was 51.6 (SD = 22.7) for the PedsQL and 74.3 (SD = 22.3) for the corresponding EORTC QLQ-C30 (n = 98). The overall mean for physical functioning of the PEDQOL was 46.8 (SD = 25.1) and the corresponding EORTC QLQ-C30 overall mean was 63.5 (SD = 27.2) (n = 156).

The correlations between the EORTC QLQ-C30 physical functioning sub-scale and the corresponding aggregate scores of the two paediatric instruments were both good, but the PedsQL physical functioning raw scores correlated more strongly (r = 0.73; 95% confidence interval (CI): 0.63, 0.81) than those of the PEDQOL (r = 0.64; CI: 0.54, 0.73). The physical functioning raw scores of the paediatric questionnaires showed only moderate agreement with those of the EORTC QLQ-C30 before linking, with similar values for the PedsQL (Lin's ρ = 0.49; CI: 0.63, 0.81) and the PEDQOL (Lin's ρ = 0.53; CI: 0.43, 0.63). Given a substantial overlap in item content, we linked the respective aggregate physical functioning scores of the PedsQL and the PEDQOL questionnaires to their EORTC QLQ-C30 equivalent.

#### 2.3.3 Linking design

To produce physical functioning crosswalks (score conversion tables), we linked scores of those participants who had completed one of the two paediatric instruments and the EORTC QLQ-C30 at the same time point. This group consisted of participants who were 16–18 years old. This linking technique, referred to as the single-group design, is akin to a repeated measures design with a single group and two treatments [31]. It is considered the most valid linking design because the scores of identical individuals are linked, thus requiring the smallest sample size to achieve the same level of accuracy as designs with a lesser degree of group equivalency [32].

To ensure that the instruments to be linked showed sufficient conceptual congruity [2], we employed two methods, modelling our approach on Choi *et al.* (2014) and Marrie *et al.* (2020). First, we reviewed the content of the physical functioning items of the three instruments to ensure that they indeed measure approximately the same concept. Second, to assess internal consistency, we calculated Cronbach's α for the three questionnaires.

#### 2.3.4 Linking function

**Fig. 2:**
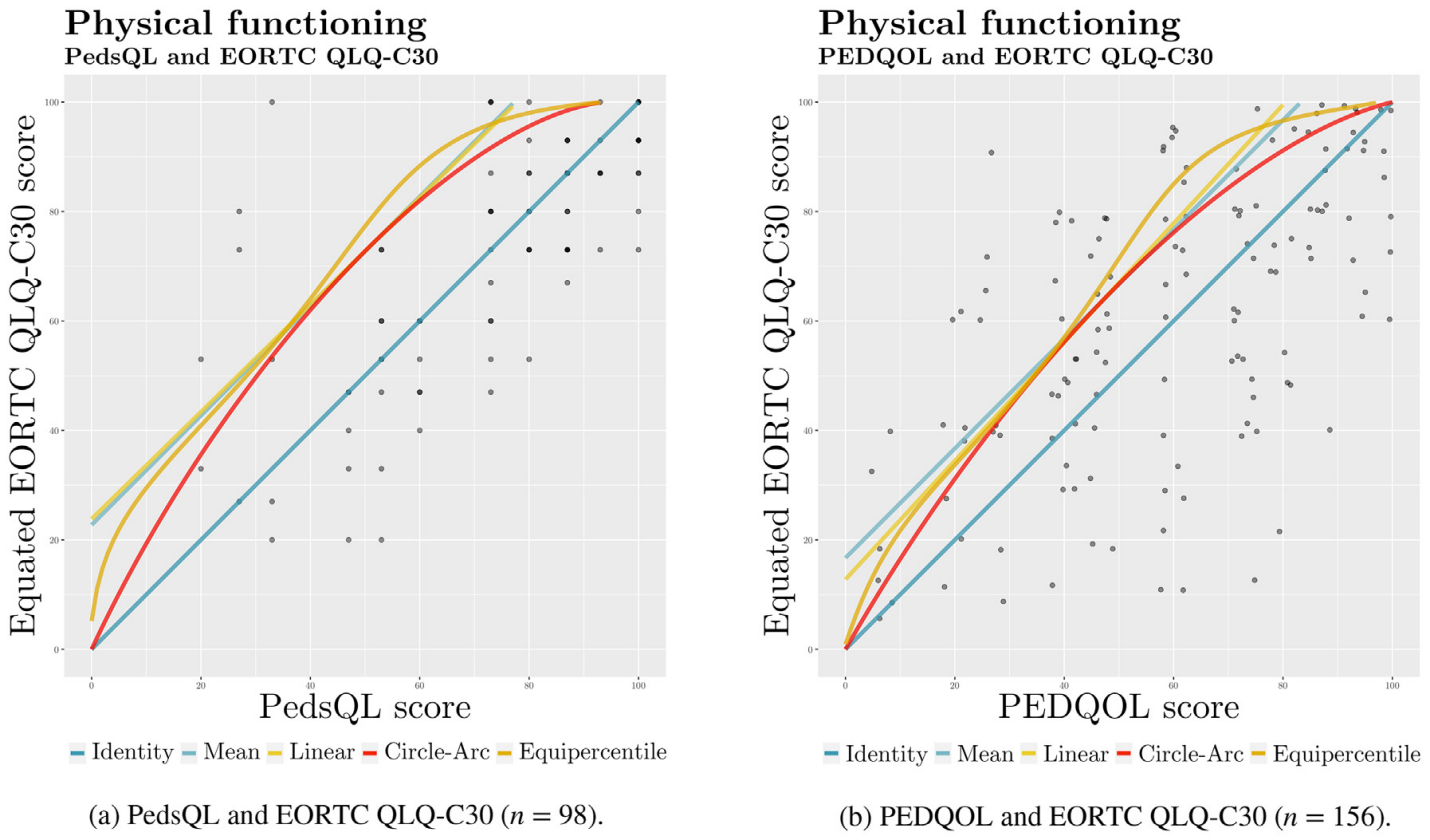
Five functions linking physical functioning scores.

We performed identity, mean, linear, equipercentile and circle-arc linking procedures (Fig. 2). Previously, we had applied log-linear pre-smoothing to three moments to adjust for potential sampling error introduced by uneven score distributions [33]. Log-linear pre-smoothing is a recommended procedure for small samples such as ours because a smoothed distribution yields more reliable results [33]. We used root mean square error (RMSE) by means of parametric bootstrapping to determine the best linking method (for details see [34], 5.7).

We chose the equipercentile linking method to produce crosswalk tables, as it emerged as the method with the most favourable linking quality parameters, overall.

#### 2.3.5 Evaluation of linking quality

**Fig. 3:**
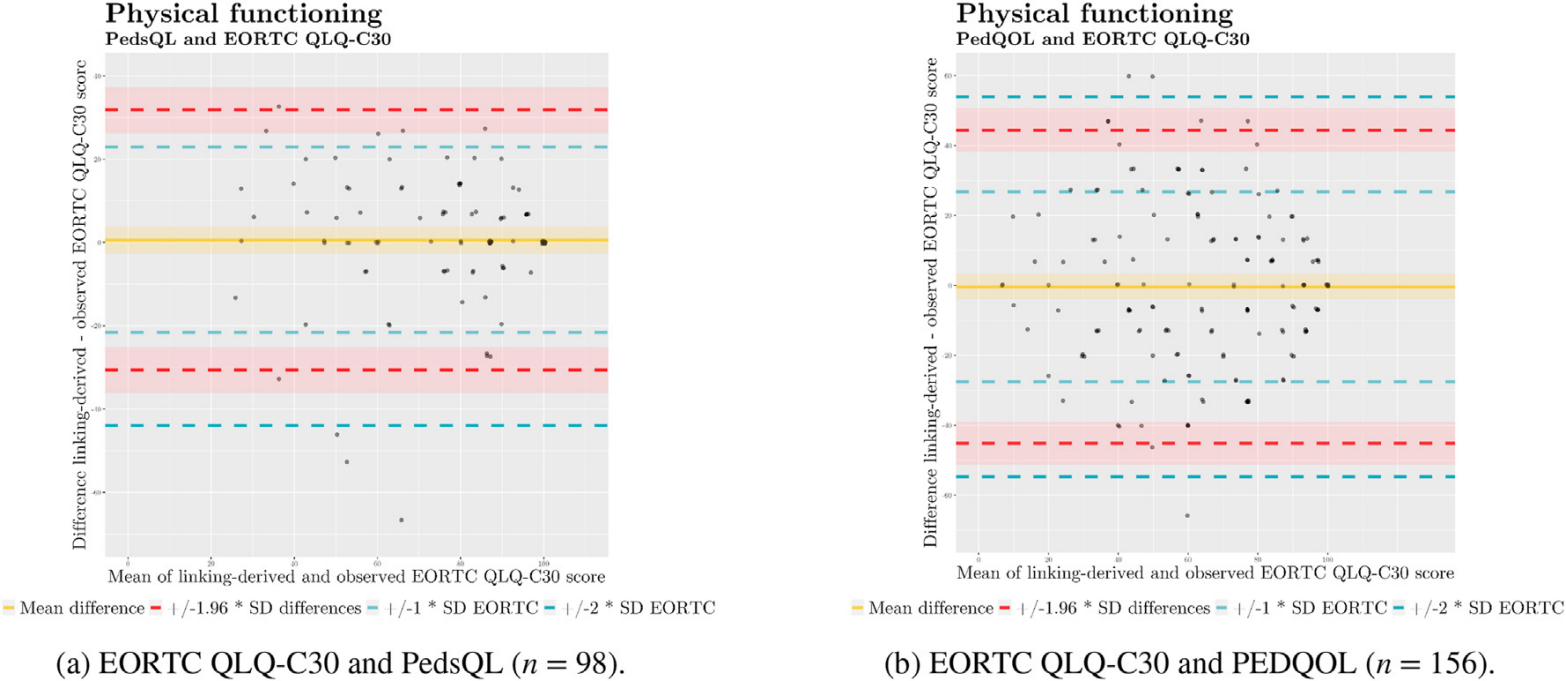
Bland–Altman plots for linked vs. observed physical functioning scores.

As a first step towards ascertaining the agreement between paediatric and adult QoL instruments, we created Bland–Altman plots [35] (Fig. 3 and Table 2d). We plotted the differences (y-axis) for scores linked from each paediatric questionnaire and those measured by the EORTC QLQ-C30 against subject means (x-axis) to check for patterns and distributions. Following Zhou *et al.* [36], we established that the limits of agreement for linked and measured scores were to be considered ”good” if they fell within one standard deviation (SD) of the mean of measured EORTC QLQ-C30 scores, ”fair” if they did not extend beyond two SDs, and ”poor”, otherwise.

**Table 2d:** Bland–Altman plots: descriptive characteristics.

Bland–Altman analysis					
	Parameter	Count	Value	*SD*	95% CI
PedsQL	Bias	98	0.53	15.92	(−2.66, 3.72)
	Upper LOA	98	31.73		(26.26, 37.21)
	Lower LOA	98	−30.67		(−36.15, −25.20)
PedQOL	Bias	156	−0.32	22.84	(−3.94, 3.29)
	Upper LOA	156	44.44		(38.25, 50.62)
	Lower LOA	156	−45.09		(−51.27, −38.90)

Additionally, we calculated Pearson's correlation coefficient *r* and Lin's concordance correlation coefficient between each of the two paediatric measures and the EORTC QLQ-C30.

**Fig. 4:**
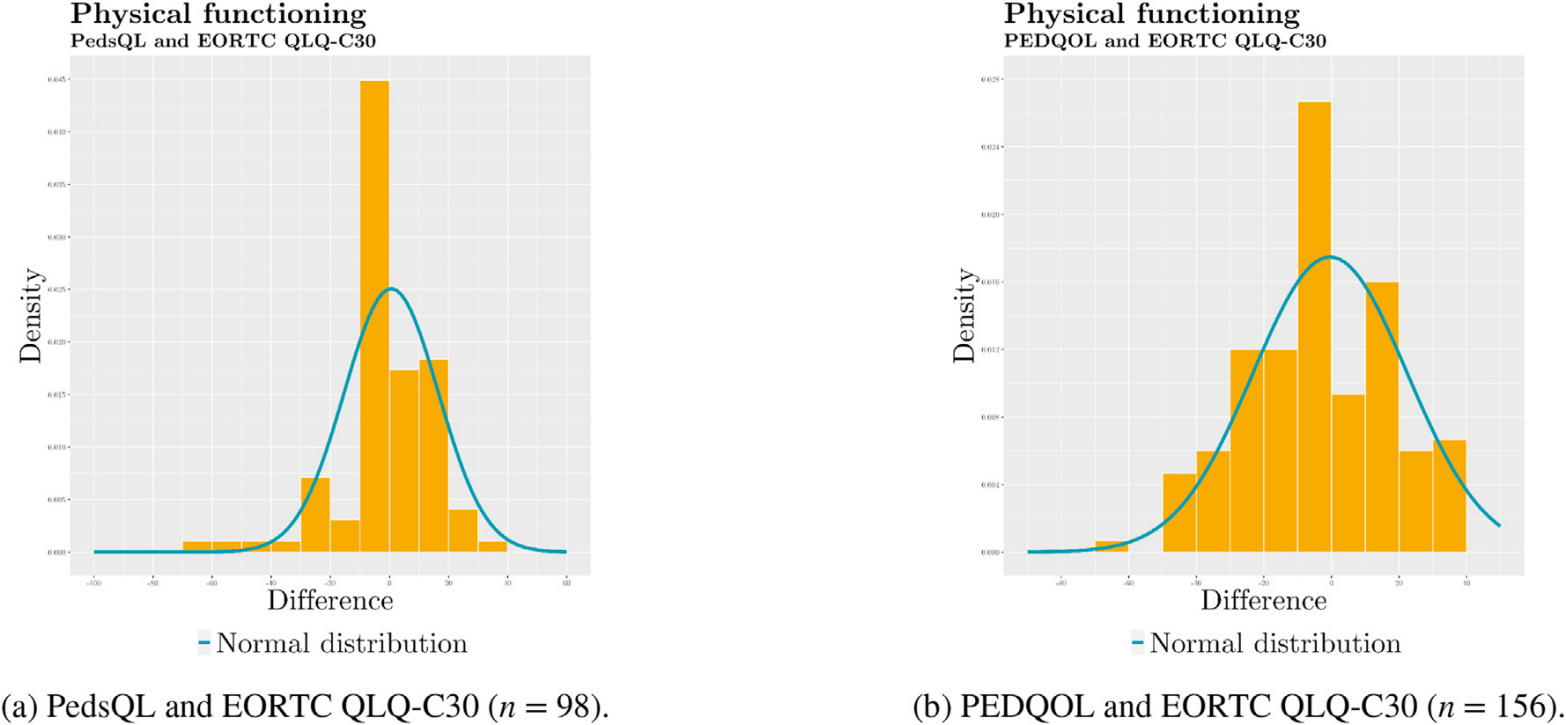
Histograms with distributions of differences between *physical functioning* scores.

We prepared histograms of the differences between measured and linked EORTC QLQ-C30 scores to visually inspect whether the distributions approximate normality (Fig. 4).

Details on software are given in Appendix A.

## 3 Results

### 3.1 Participant characteristics

The QoL sub-sample consisted of 2213 osteosarcoma patients. The mean age at registration was 15.1 (*SD* = 5.3) years. Out of the complete sub-sample, 760 participants had completed the PedsQL in the *physical functioning* domain at one or more of the four time points, and 337 had completed the PEDQOL in this domain at one time point or more. Out of these participants, 75 participants between the ages of 16 and 18 had completed both the PedsQL and the EORTC QLQ-C30 in the *physical functioning* domain at the same time point on 98 occasions, and 112 had completed both the PEDQOL and the EORTC QLQ-C30 concurrently on 156 occasions.

Table 3a gives an overview of patient characteristics by linked questionnaire (PedsQL or PEDQOL) for the *physical functioning* domain, including sex, age, and study group, overall and by linked sub-sample.

**Table 3a:** Characteristics of participants in the physical functioning domain by paediatric questionnaire, overall and linked.

Characteristics	Physical functioning^1^			
	PedsQL		PEDQOL	
	Overall	Linked to EORTC QLQ-C30	Overall	Linked to EORTC QLQ-C30
**Sex, n(%)**				
Male	429 (56)	48 (64)	171 (51)	68 (61)
Female	331 (44)	27 (36)	164 (49)	44 (39)
**Age (years)**^2^				
**Age group, n(%)**				
5 to 15	671 (88)	44 (59)	275 (82)	70 (62)
16 to 17	74 (10)	22 (29)	59 (18)	42 (38)
18 or older	15 (2)	9 (12)	1 (0)	0 (0)
Mean (SD)	12.8 (3.0)	15.1 (2.7)	13.4 (2.9)	15.6 (1.3)
**Study group, n(%)**				
COG	616 (81)	96 (98)	0 (0)	0 (0)
COSS	0 (0)	0 (0)	211 (63)	83 (74)
EOI	144 (19)	2 (2)	59 (18)	22 (20)
SSG	0 (0)	0 (0)	65 (19)	7 (6)

COG: Childrens's Oncology Group; COSS: Cooperative Osteosarcoma Group; EOI: European Osteosarcoma Intergroup; SSG: Scandinavian Sarcoma Group.

1 The columns pertain to those participants whose PedsQL or PEDQOL scores were linked to their respective EORTC QLQ-C30 scores. Therefore, the table does not contain a separate column for EORTC QLQ-C30 scores.

2 Age refers to the age at the time of registration for participation in the study.

#### 3.1.1 Bland–Altman plots

We used Bland–Altman plots to compare PedsQL and PEDQOL scores to EORTC QLQ C-30 scores. The interpretation of Bland–Altman plots is premised on normality and homoscedasticity of the distribution. We prepared histograms for the distributions of differences (Fig. 4 and Table 2d) to make a first visual assessment. We then prepared Bland–Altman plots (Fig. 3) displaying the differences in scores between each paediatric instrument and the EORTC QLQ-C30 against the respective means.

**Fig. 5:**
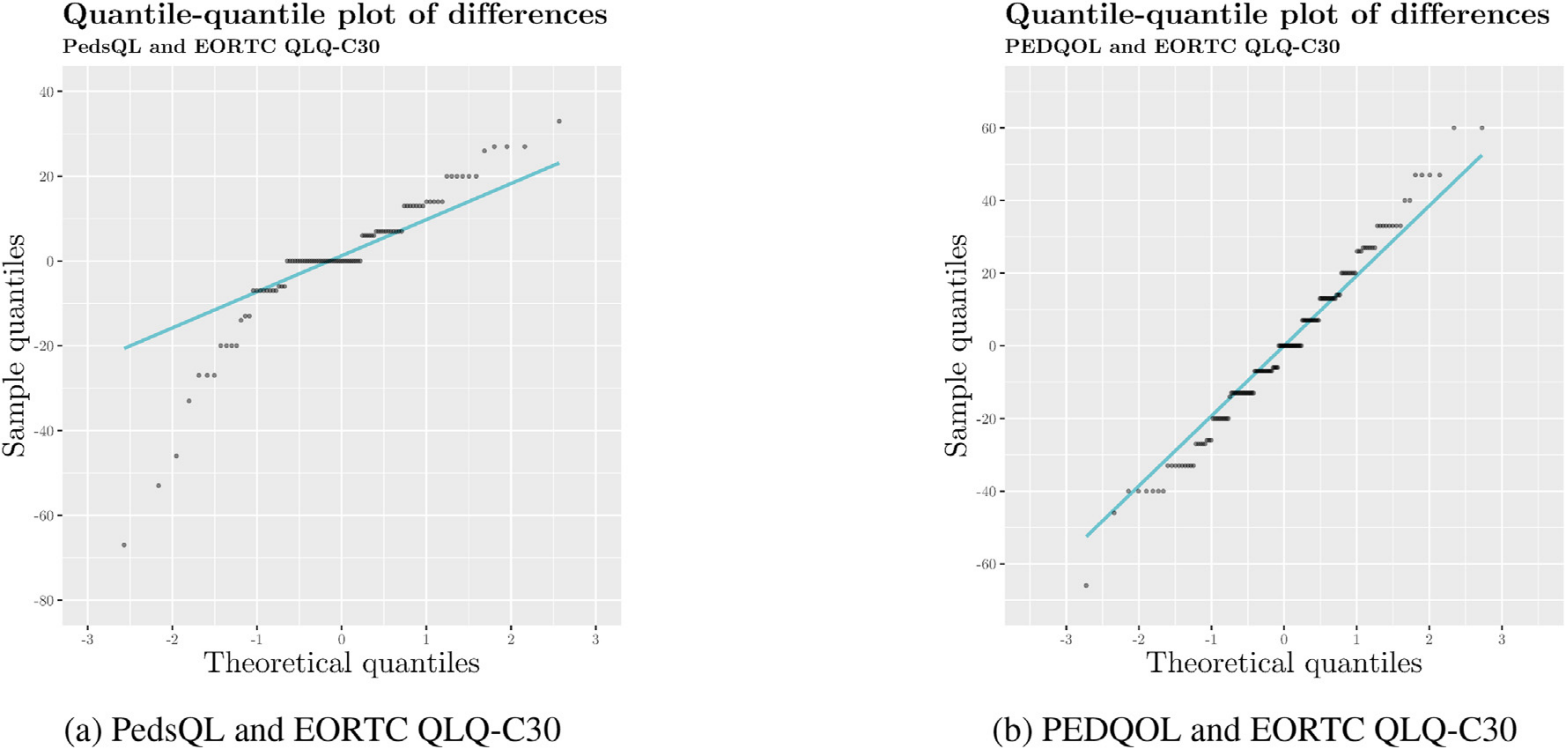
Quantile–quantile plots of differences between physical functioning scores.

**Fig. 6:**
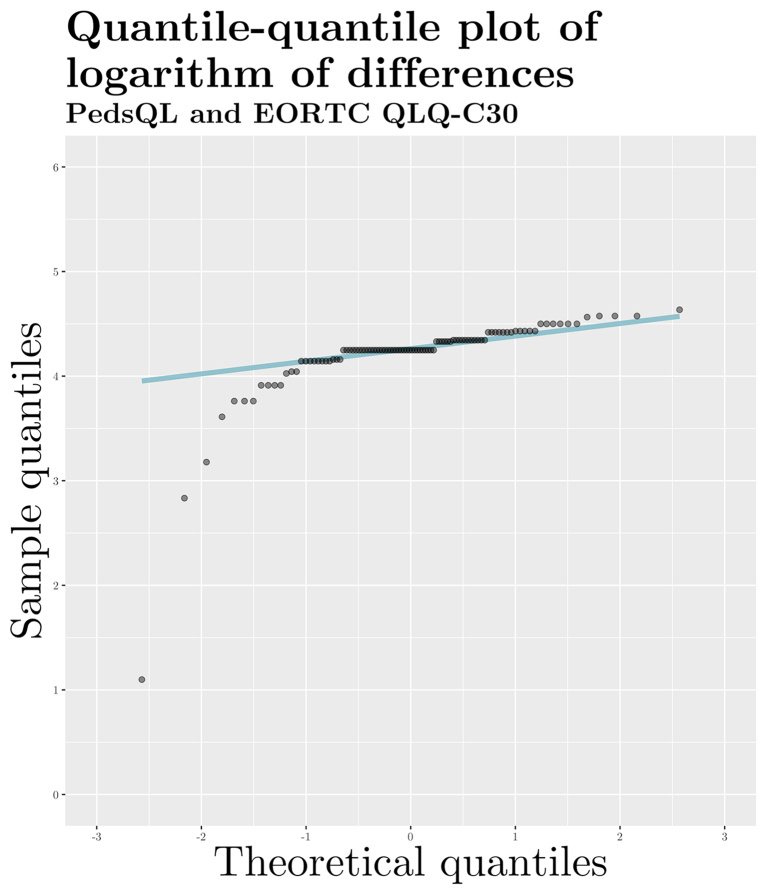
Quantile–quantile plot of logarithm of differences between PedsQL and EORTC QLQ-C30 physical functioning scores (n = 98).

**Fig. 7:**
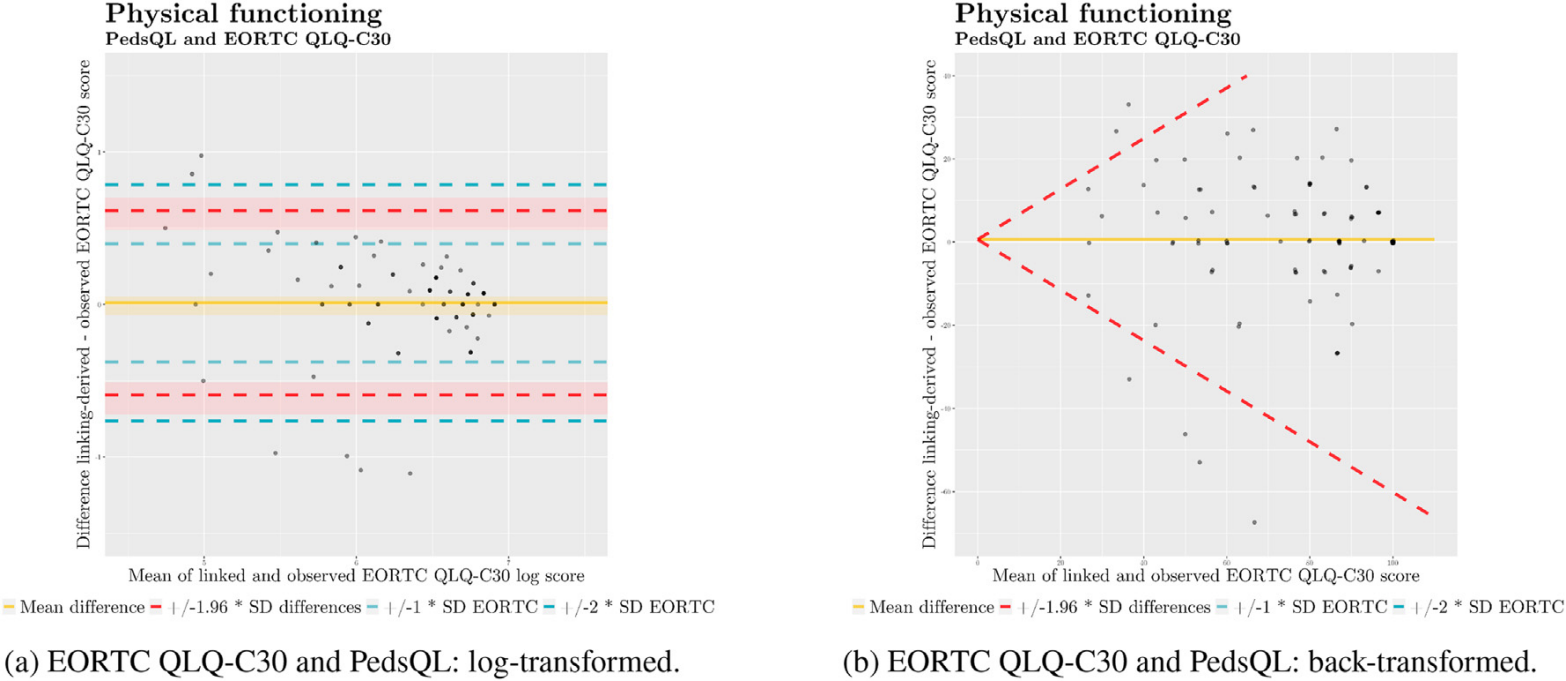
Bland–Altman plots for linked vs. observed log-transformed and back-transformed physical functioning scores (n = 98).

To inspect for heteroscedasticity, we prepared quantile–quantile (Q–Q) plots (Fig. 5) for differences between scores linked from the two paediatric questionnaires and EORTC QLQ-C30 scores. We judged that scores linked from the PEDQOL displayed adequate homoscedasticity. However, scores linked from the PedsQL indicated an uneven, left-skewed distribution. Therefore, we log-transformed the score differences, achieving better overall homoscedasticity, albeit with a remaining left skew (Fig. 6). To account for the presence of substantial heteroscedasticity in scores linked from the PedsQL, we prepared a Bland–Altman plot on log-transformed data (Fig. 7a) which indicated a better fit of limits of agreement. Given that log-transformed scores do not lend themselves to easy interpretation for clinical practice, we additionally plotted the score differences in a conventional Bland–Altman plot on the original scale with back-transformed limits of agreement (Fig. 7b) [37,38].

Summarily, we judged agreement for physical functioning scores acceptable, as the limits of agreement did not extend beyond two standard deviations of EORTC QLQ-C30 scores for either of the paediatric instruments, and the majority of scores being within one standard deviation of EORTC QLQ-C30 scores.

#### 3.1.2 Correlations between physical functioning aggregate scores of paediatric and adult instruments

Additionally, we calculated Pearson's r and Lin's ρ− [39] concordance correlation coefficients between the EORTC QLQ-C30 and the PedsQL and PEDQOL physical functioning converted scores.

The correlation coefficients for physical functioning scores were good for both the PedsQL and the PEDQOL to EORTC QLQ-C30 conversions, with a Lin's ρ of 0.74 and 0.64, respectively (Table 3b and 3c).

**Table 3b:** Paediatric questionnaires and EORTC QLQ-C30: Correlation and concordance coefficients post-linking.

Coefficient	Physical functioning	
	PedsQL	PEDQOL
Pearson's *r* (95% CI)	0.74 (0.64–0.82)	0.64 (0.54–0.72)
Lin's *ρ* (95% CI)	0.74 (0.64–0.82)	0.64 (0.54–0.72)

**Table 3c:** Interpretation of concordance correlation coefficients [35].

Concordance Correlation coefficient	Strength of agreement
<0.20	Poor
0.21–0.40	Fair
0.41–0.60	Moderate
0.61–0.80	Good
0.81–1.00	Very good

#### 3.1.3 Correlations between other aggregate scores of paediatric and adult instruments

The converted scores of the PedsQL and PEDQOL *fatigue* both correlated well with EORTC QLQ-C30 scores (Lin's *ρ* = 0.69 and Lin's *ρ* = 0.71). Correlation coefficients for *pain* were moderate for the PedsQL (Lin's *ρ* = 0.58) and good for the PEDQOL (Lin's *ρ* = 0.73). Correlation coefficients for *emotional functioning* were moderate (Lin's *ρ* = 0.55) for the PedsQL and fair for PEDQOL (Lin's *ρ* = 0.36) conversions to EORTC QLQ-C30 scores. The correlation of converted *cognitive functioning* scores with EORTC QLQ-C30 scores was fair for the PedsQL (Lin's *ρ* = 0.37) and moderate for the PEDQOL (Lin's *ρ* = 0.47). Converted *social functioning* scores correlated poorly with EORTC QLQ-C30 scores for both, the PedsQL (Lin's *ρ* = 0.17) and the PEDQOL PedsQL (Lin's *ρ* = 0.08).

## 4 Discussion

Data harmonisation provides a number of benefits by permitting the pooling of data, such as answering novel research questions or increasing statistical power. Despite a growing interest in harmonising data, retrospective data harmonisation (after data collection) is the rule and prospective harmonisation (before data collection) the exception [3]. While it may be due to a lack of foresight or practicability that retrospective data harmonisation remains the only option, harmonising data prospectively may also be inherently impossible. This was the case in the international research collaboration the present study grew out of which included longitudinal QoL assessments in adult survivors of childhood osteosarcoma. The use of different PRO measures during childhood and adulthood was unavoidable, as no suitable instrument for both age groups existed.

To obtain harmonised data retrospectively, we linked the scores from two paediatric PRO measures to an adult PRO measure to assess the quality of life across the lifespan of osteosarcoma survivors. Visual and numerical concordance assessments indicated good agreement between physical functioning aggregate scores. The equipercentile linking method yielded the best overall results for this sample. Sub-sets consisting of 75 (PedsQL) and 112 participants (PEDQOL) yielded 98 (PedsQL) and 156 (PEDQOL) score pairings between paediatric and adult questionnaires and were sufficient to permit score linking for the whole cohort and enabled the analysis of QoL data for a forthcoming publication.

In domains other than *physical functioning*, the concordance estimates obtained with Pearson's *r* diverged from those obtained with dedicated concordance coefficients (Appendix, Table D.1), thus confirming that Pearson's *r* is not a useful measure for assessing intra-individual agreement. The Pearson correlation coefficient (Pearson's *r*) is generally not considered a suitable measure of concordance because it is only informative if the relationship between two variables is linear, thus potentially leading to incorrect conclusions in case of non-linearity. Crucially, Pearson's *r* only evaluates the extent of a linear relationship on a population level, ignoring intra-individual concordance. Despite its apparent shortcomings, Pearson's *r* continues to be widely employed in the score linking literature as a measure of agreement between two instruments. This is all the more surprising, given that non-linear score linking methods were presumably developed to specifically account for non-linear agreement between two instruments. Due to its continued popularity and to underscore differences between concordance measures, we nevertheless included Pearson's *r* alongside Lin's concordance correlation coefficient *ρ* [39] which we consider more apt. We provide an evaluation according to value ranges to allow a verbal interpretation, similar to the kappa concordance coefficient for binary variables [35], with five categories, ranging from ”Poor” to ”Very Good” (Table 3c).

Building on McNemar's coefficient of alienation, Dorans [40] defined ”Reduction in Uncertainty”. Since a 50% reduction in uncertainty, as measured in score units, requires a Pearson's *r* of at least 0.866, Dorans recommended a correlation of this magnitude as an appropriate lower bound. This recommendation was made in the context of high-stakes educational testing, as Choi and colleagues [12] have pointed out. For linking health outcome measures, they suggesteda correlation of 0.75–0.80 as an appropriate minimum, given that aggregate outcomes are the focus of interest, and in particular when using a single-group design which permits the direct evaluation of accuracy.

A limitation of our study is that our results may not be population invariant, i.e. the linking quality parameters we obtained may not generalise to other populations. Previous studies linking PedsQL or PEDQOL *physical functioning* aggregate scores to the EORTC QLQ-C30 are lacking. Therefore, we were unable to draw comparisons to similar or dissimilar populations and we cannot generalise our findings beyond the highly selective clinical population our sample was drawn from. The aim of our study was to evaluate the feasibility of linking paediatric and adult PRO measures within a population of osteosarcoma survivors. Clearly, our findings are restricted to this narrowly circumscribed area of clinical practice and research. The methodology also does not allow for harmonisation in completely disparate age groups (e.g. 5-10 year-old with 35–40 year-old).

The use of age-adequate (i.e. age-specific) questionnaires for children seems unavoidable, rendering a direct comparison of paediatric and adult scores in survivors of childhood cancer inherently impossible. Therefore, we see the potential general utility of score linking in this field in offering interoperability of paediatric and adult PRO measures, and the specific value of this study in showing the viability of this approach for the first time. Having established its feasibility, the approach described may be integrated in future study designs involving dissimilar populations. Doing so may yield evidence regarding the population invariance of our results.

Another limitation of our study is that we cannot rule out an order effect, i.e. the relationship of the instruments may have depended on the order of their administration. This point should be addressed in future investigations by randomising the order of administration. In a similar vein, the administration of two questionnaires at the same point may have biased the responses to the second questionnaire. Randomising the order of administration should also reduce fatigue bias, by equalising the directionality of such an effect between the instruments.

We consider the single-group design a major strength of our study, as it provides the firmest methodological grounds for score linking. Its inherent potential disadvantages should be balanced against its strengths and against the weaknesses of alternative linking designs. Using a single-group design, we obtained actual and linking-derived scores from the same population. This allowed us to evaluate the accuracy of our linking functions directly. As a tangible product, we created crosswalk tables between PedsQL and PEDQOL *physical functioning* aggregate scores (see Appendix, Table L.1) which will bring forward data harmonisation and will enable us to perform longitudinal analyses within the EURAMOS-1 cohort.

With score linking, it is possible to directly compare scores of osteosarcoma patients obtained with distinct age-group-specific inventories and observe their QoL across the entire lifespan. The approach may create the conditions for conducting longitudinal mixed-model meta-analyses. We consider score linking a promising tool for assuring comparability of intra-individual QoL assessments in studies over time and extending across different stages of life. We anticipate that oncological QoL research may strongly benefit from score linking.

## Funding

The study sponsor was the UK 10.13039/501100000265Medical Research Council in Europe and the US 10.13039/100000054National Cancer Institute in North America and Australia. Each trial group organised local coordination elements; central coordination and analysis was led from Medical Research Council Clinical Trials Unit at University College of London. Neither the sponsors nor the funders of the trial had a role in trial design, data analysis, or data interpretation. The EURAMOS-1 is an academic clinical trial funded through multiple national and international government agencies and cancer charities: - Children's Oncology Group funding for the EURAMOS-1 trial (AOST0331) was supported by the National Clinical Trial Network (NCTN) Operations Centre Grant U10CA180886, NCTN Statistics and Data Center Grant U10CA180899 and St. Baldrick's Foundation. - 10.13039/501100000782European Science Foundation under the European Science Foundation Collaborative Research Programme for Pan-European Clinical Trials, through contract number ERASCT-2003-980409 of the European Commission, DG Research, FP6 (Ref No MM/NG/EMRC/0202) National funding in Europe was provided by the following:

⋅ Belgium: Fonds National de la Recherche Scientifique Belgium FWO (Fonds voor Wetenschappelijk Onderzoek-Vlaanderen)
⋅ Denmark: Danish Medical Research Council
⋅ Finland: Academy of Finland
⋅ Germany: Deutsche Forschungsgemeinschaft ref No: BI 1045/1-1 & 1–2, Deutsche Krebshilfe (DKH) ref No: 50-2723-Bi2
⋅ Hungary: Semmelweis Foundation
⋅ Netherlands: ZonMw (Council for Medical Research)
⋅ Norway: Research Council of Norway
⋅ Sweden: SSG and Swedish Childhood Cancer Fund
⋅ Switzerland: Swiss Paediatric Oncology Group
⋅ United Kingdom: includes funding for the trial coordinating data centre (MRC Clinical Trials Unit at UCL): Cancer Research UK, CRUK/05/013, Medical Research Council: MC_UU_12023/28.

Additional funding to the University of Münster Centre for Clinical Trials, site of the EURAMOS Intergroup Safety Desk: 10.13039/501100002347Federal Ministry of Education and Research, Germany, BMBF 01KN1105.

## CRediT authorship contribution statement

**Axel Budde:** Conceptualisation, Data curation, Formal analysis, Methodology, Software, Writing - original draft preparation, Visualisation. **Katja Baust:** Conceptualisation, Data curation, Writing - review and editing, Project administration. **Leonie Weinhold:** Methodology, Validation, Writing - review & editing. **Mark Bernstein:** Investigation, Writing - review and editing. **Stefan Bielack:** Investigation, Writing - review and editing. **Catharina Dhooge:** Investigation, Writing - review and editing. **Lars Hjorth:** Investigation, Writing - review and editing. **Katherine A. Janeway:** Investigation, Writing - review and editing. **Meriel Jenney:** Investigation, Writing - review and editing. **Mark D. Krailo:** Investigation, Writing - review and editing. **Neyssa Marina:** Investigation, Writing - review and editing. **Rajaram Nagarajan:** Investigation, Writing - review and editing. **Sigbjørn Smeland:** Investigation, Writing - review and editing. **Matthew R. Sydes:** Investigation, Methodology, Writing - reviewand editing. **Patricia DeVos:** Investigation, Writing - review and editing. **Jeremy Whelan:** Investigation, Writing - review and editing. **Andreas Wiener:** Investigation, Writing - review and editing. **Gabriele Calaminus:** Conceptualisation, Investigation, Writing - review and editing, Supervision. **Matthias Schmid:** Conceptualisation, Methodology, Writing - review and editing, Supervision.

## Conflict of interest statement

The authors declare the following financial interests/personal relationships which may be considered as potential competing interests: SB reports grants from Deutsche Krebshilfe, Deutsche Forschungsgemeinschaft, and European Science Foundation during the conduct of the study and personal fees from Lilly, Bayer, Pfizer, Novartis, Isofol, Clinigen, Sensorion, Ipsen, and Roche outside the submitted work. MRS reports grants and nonfinancial support from Astellas, grants from Clovis, grants and nonfinancial support from Janssen, grants and nonfinancial support from Novartis, grants and nonfinancial support from Pfizer, and grants and nonfinancial support from Sanofi, during the conduct of the study and personal fees from Lilly Oncology and personal fees from Janssen for educational courses and workshops outside the submitted work. NM reports employment by Five Prime Therapeutics, Inc and Sanofi US, outside the submitted work. The remaining authors declare no conflicts of interest.

## Figures and Tables

**Table B.1:** Characteristics of PedsQL participants by domain, overall and linked.

Characteristics	PedsQL participants by domain											
	Functional								Symptom			
	Physical Functioning		Emotional Functioning		Cognitive Functioning		Social Functioning		Fatigue		Pain	
	Overall	Linked	Overall	Linked	Overall	Linked	Overall	Linked	Overall	Linked	Overall	Linked
**Sex, *n*(%)**												
Male	429 (56)	48 (64)	445 (57)	51 (64)	395 (57)	45 (64)	439 (56)	45 (64)	445 (57)	45 (64)	443 (57)	48 (62)
Female	331 (44)	27 (36)	339 (43)	29 (36)	296 (43)	25 (36)	339 (44)	25 (36)	341 (43)	25 (36)	341 (44)	29 (38)
**Age (years)**^1^												
**Age group, *n*(%)**												
5 to 15	671 (88)	44 (59)	690 (88)	48 (60)	609 (88)	43 (61)	685 (88)	43 (61)	694 (88)	43 (61)	692 (88)	47 (61)
16 to 17	74 (10)	22 (29)	78 (10)	30 (29)	69 (10)	20 (29)	77 (10)	20 (29)	76 (10)	20 (29)	76 (10)	21 (27)
18 or older	15 (2)	9 (12)	16 (2)	7 (11)	13 (2)	9 (10)	16 (2)	7 (10)	16 (2)	7 (10)	16 (2)	9 (12)
Mean(*SD*)	12.8 (3.0)	15.1 (2.7)	12.8 (3.0)	15.1 (2.6)	12.8 (3.0)	15.0 (2.6)	12.8 (3.0)	15.0 (2.6)	12.8 (3.0)	15.0 (2.6)	12.8 (3.0)	15.1 (2.7)
**Study group, *n*(%)**												
COG	616 (81)	73 (97)	640 (82)	78 (98)	562 (81)	68 (97)	632 (81)	68 (97)	639 (81)	68 (97)	638 (81)	75 (97)
COSS	0 (0)	0 (0)	0 (0)	0 (0)	0 (0)	0 (0)	0 (0)	0 (0)	0 (0)	0 (0)	0 (0)	0 (0)
EOI	144 (19)	2 (3)	144 (18)	2 (2)	129 (19)	2 (3)	146 (19)	2 (3)	147 (19)	2 (3)	146 (19)	2 (3)
SSG	0 (0)	0 (0)	0 (0)	0 (0)	0 (0)	0 (0)	0 (0)	0 (0)	0 (0)	0 (0)	0 (0)	0 (0)

COG: Childrens's Oncology Group; COSS: Cooperative Osteosarcoma Group; EOI: European Osteosarcoma Intergroup; SSG: Scandinavian Sarcoma Group.

1 Age refers to the age at the time of registration for participation in the study.

**Table B.2:** Characteristics of PEDQOL participants by domain, overall and linked.

Characteristics	PedsQL participants by domain											
	Functional								Symptom			
	Physical Functioning		Emotional Functioning		Cognitive Functioning		Social Functioning		Fatigue		Pain	
	Overall	Linked	Overall	Linked	Overall	Linked	Overall	Linked	Overall	Linked	Overall	Linked
**Sex, *n*(%)**												
Male	429 (56)	48 (64)	445 (57)	51 (64)	395 (57)	45 (64)	439 (56)	45 (64)	445 (57)	45 (64)	443 (57)	48 (62)
Female	331 (44)	27 (36)	339 (43)	29 (36)	296 (43)	25 (36)	339 (44)	25 (36)	341 (43)	25 (36)	341 (44)	29 (38)
**Age (years)**^1^												
**Age group, *n*(%)**												
5 to 15	671 (88)	44 (59)	690 (88)	48 (60)	609 (88)	43 (61)	685 (88)	43 (61)	694 (88)	43 (61)	692 (88)	47 (61)
16 to 17	74 (10)	22 (29)	78 (10)	30 (29)	69 (10)	20 (29)	77 (10)	20 (29)	76 (10)	20 (29)	76 (10)	21 (27)
18 or older	15 (2)	9 (12)	16 (2)	7 (11)	13 (2)	9 (10)	16 (2)	7 (10)	16 (2)	7 (10)	16 (2)	9 (12)
Mean(*SD*)	12.8 (3.0)	15.1 (2.7)	12.8 (3.0)	15.1 (2.6)	12.8 (3.0)	15.0 (2.6)	12.8 (3.0)	15.0 (2.6)	12.8 (3.0)	15.0 (2.6)	12.8 (3.0)	15.1 (2.7)
**Study group, *n*(%)**												
COG	616 (81)	73 (97)	640 (82)	78 (98)	562 (81)	68 (97)	632 (81)	68 (97)	639 (81)	68 (97)	638 (81)	75 (97)
COSS	0 (0)	0 (0)	0 (0)	0 (0)	0 (0)	0 (0)	0 (0)	0 (0)	0 (0)	0 (0)	0 (0)	0 (0)
EOI	144 (19)	2 (3)	144 (18)	2 (2)	129 (19)	2 (3)	146 (19)	2 (3)	147 (19)	2 (3)	146 (19)	2 (3)
SSG	0 (0)	0 (0)	0 (0)	0 (0)	0 (0)	0 (0)	0 (0)	0 (0)	0 (0)	0 (0)	0 (0)	0 (0)

COG: Childrens's Oncology Group; COSS: Cooperative Osteosarcoma Group; EOI: European Osteosarcoma Intergroup; SSG: Scandinavian Sarcoma Group.

1 Age refers to the age at the time of registration for participation in the study.

**Table C.1:** Internal consistency reliability of the *emotional functioning* aggregate scores of the three instruments.

Time point	Questionnaire	Linked to	*N*	Cronbach's *α* (95% CI)	Item–total correlation		
					Min	Mean	Max
**E1**	**PedsQL**	EORTC QLQ-C30	39	0.74 (0.61, 0.87)	0.36	0.62	0.82
	**PEDQOL**		47	0.56 (0.70, 0.84)	0.31	0.60	0.79
	**EORTC QLQ-C30**	PedsQL	39	0.72 (0.82, 0.91)	0.52	0.72	0.91
		PEDQOL	47	0.73 (0.82, 0.90)	0.65	0.71	0.80
**E2**	**PedsQL**	EORTC QLQ-C30	29	0.72 (0.82, 0.93)	0.44	0.71	0.80
	**PEDQOL**		51	0.61 (0.73, 0.85)	0.37	0.63	0.77
	**EORTC QLQ-C30**	PedsQL	29	0.73 (0.82, 0.92)	0.56	0.73	0.85
		PEDQOL	51	0.72 (0.81, 0.90)	0.55	0.71	0.81
**E3**	**PedsQL**	EORTC QLQ-C30	19	0.60 (0.77, 0.93)	0.55	0.65	0.79
	**PEDQOL**		43	0.35 (0.56, 0.77)	0.38	0.48	0.75
	**EORTC QLQ-C30**	PedsQL	19	0.60 (0.77, 0.95)	0.59	0.73	0.87
		PEDQOL	43	0.67 (0.78, 0.88)	0.55	0.68	0.82
**E4**	**PedsQL**	EORTC QLQ-C30	18	0.66 (0.81, 0.95)	0.29	0.70	0.87
	**PEDQOL**		32	0.45 (0.65, 0.85)	0.39	0.56	0.76
	**EORTC QLQ-C30**	PedsQL	18	0.55 (0.73, 0.91)	0.37	0.63	0.77
		PEDQOL	32	0.65 (0.77, 0.90)	0.48	0.67	0.73

**Table C.2:** Internal consistency reliability of the *cognitive functioning* aggregate scores of the three instruments.

Time point	Questionnaire	Linked to	*N*	Cronbach's *α* (95% CI)		Item–total correlation		
						Min	Mean	Max
**E1**	**PedsQL**	EORTC QLQ-C30	33	0.47	(0.14, 0.80)	0.47	0.47	0.47
	**PEDQOL**		36	0.76	(0.63, 0.88)	0.41	0.63	0.76
	**EORTC QLQ-C30**	PedsQL	33	0.06	(-0.56, 0.68)	0.13	0.13	0.13
		PEDQOL	38	0.66	(0.45, 0.88)	0.61	0.61	0.61
**E2**	**PedsQL**	EORTC QLQ-C30	18	0.56	(0.18, 0.94)	0.54	0.54	0.54
	**PEDQOL**		33	0.68	(0.50, 0.85)	0.36	0.54	0.68
	**EORTC QLQ-C30**	PedsQL	18	0.21	(-0.47, 0.88)	0.27	0.27	0.27
		PEDQOL	33	0.41	(0.01, 0.81)	0.41	0.41	0.41
**E3**	**PedsQL**	EORTC QLQ-C30	19	0.80	(0.62, 0.97)	0.75	0.75	0.75
	**PEDQOL**		41	0.84	(0.77, 0.92)	0.53	0.72	0.81
	**EORTC QLQ-C30**	PedsQL	19	0.62	(0.36, 0.87)	0.66	0.66	0.66
		PEDQOL	41	0.33	(-0.07.0.74)	0.35	0.35	0.35
**E4**	**PedsQL**	EORTC QLQ-C30	16	0.94	(0.89, 1.00)	0.92	0.92	0.92
	**PEDQOL**		32	0.74	(0.60, 0.88)	0.54	0.62	0.76
	**EORTC QLQ-C30**	PedsQL	16	0.04	(-0.88, 0.97)	0.11	0.11	0.11
		PEDQOL	41	0.33	(-0.07, 0.74)	0.69	0.69	0.69

**Table C.3:** Internal consistency reliability of the *social functioning* aggregate scores of the three instruments.

Time point	Questionnaire	Linked to	*N*	Cronbach's *α* (95% CI)		Item–total correlation		
						Min	Mean	Max
**E1**	**PedsQL**	EORTC QLQ-C30	38	0.34	(−0.01, 0.69)	0.17	0.42	0.65
	**PEDQOL**		47	0.24	(−0.09, 0.58)	0.05	0.30	0.53
	**EORTC QLQ-C30**	PedsQL	38	0.66	(0.45, 0.87)	0.61	0.61	0.61
		PEDQOL	47	0.67	(0.49, 0.86)	0.62	0.62	0.62
**E2**	**PedsQL**	EORTC QLQ-C30	27	0.62	(0.41, 0.83)	0.39	0.57	0.67
	**PEDQOL**		40	−0.22	(−0.81, 0.38)	−0.33	0.20	0.81
	**EORTC QLQ-C30**	PedsQL	27	0.61	(0.32, 0.90)	0.57	0.57	0.57
		PEDQOL	40	0.70	(0.52, 0.89)	0.65	0.65	0.65
**E3**	**PedsQL**	EORTC QLQ-C30	19	0.48	(0.14, 0.83)	−0.10	0.46	0.71
	**PEDQOL**		28	−0.27	(−1.05, 0.50)	−0.50	0.15	0.49
	**EORTC QLQ-C30**	PedsQL	19	0.79	(0.62, 0.96)	0.76	0.76	0.76
		PEDQOL	28	0.58	(0.30, 0.86)	0.57	0.57	0.57
**E4**	**PedsQL**	EORTC QLQ-C30	17	0.60	(0.36, 0.85)	0.32	0.50	0.68
	**PEDQOL**		24	−0.02	(−0.68, 0.64)	−0.03	0.21	0.71
	**EORTC QLQ-C30**	PedsQL	17	0.78	(0.58, 0.98)	0.74	0.74	0.74
		PEDQOL	24	0.89	(0.81, 0.98)	0.85	0.85	0.85

**Table C.4:** Internal consistency reliability of the *fatigue* aggregate scores of the three instruments.

Time point	Questionnaire	Linked to	*N*	Cronbach's *α* (95% CI)		Item–total correlation		
						Min	Mean	Max
**E1**	**PedsQL**	EORTC QLQ-C30	40	–	–	–	–	–
	**PEDQOL**		50	–	–	–	–	–
	**EORTC QLQ-C30**	PedsQL	40	0.82	(0.71, 0.93)	0.77	0.77	0.77
		PEDQOL	50	0.89	(0.83, 0.95)	0.85	0.85	0.85
**E2**	**PedsQL**	EORTC QLQ-C30	30	–	–	–	–	–
	**PEDQOL**		54	–	–	–	–	–
	**EORTC QLQ-C30**	PedsQL	30	0.64	(0.39, 0.90)	0.59	0.59	0.59
		PEDQOL	54	0.85	(0.77, 0.93)	0.80	0.80	0.80
**E3**	**PedsQL**	EORTC QLQ-C30	19	–	–	–	–	–
	**PEDQOL**		45	–	–	–	–	–
	**EORTC QLQ-C30**	PedsQL	19	0.87	(0.75, 0.99)	0.82	0.82	0.82
		PEDQOL	45	0.88	(0.81, 0.95)	0.83	0.83	0.83
**E4**	**PedsQL**	EORTC QLQ-C30	17	–	–	–	–	–
	**PEDQOL**		32	–	–	–	–	–
	**EORTC QLQ-C30**	PedsQL	17	0.73	(0.50, 0.97)	0.70	0.70	0.70
		PEDQOL	32	0.59	(0.35, 0.83)	0.59	0.59	0.59

**Table C.5:** Internal consistency reliability of the *pain* aggregate scores of the three instruments.

Time point	Questionnaire	Linked to	*N*	Cronbach's *α* (95% CI)		Item–total correlation		
						Min	Mean	Max
**E1**	**PedsQL**	EORTC QLQ-C30	40	–	–	–	–	–
	**PEDQOL**		50	–	–	–	–	–
	**EORTC QLQ-C30**	PedsQL	40	0.82	(0.71, 0.93)	0.77	0.77	0.77
		PEDQOL	50	0.89	(0.83, 0.95)	0.85	0.85	0.85
**E2**	**PedsQL**	EORTC QLQ-C30	30	–	–	–	–	–
	**PEDQOL**		54	–	–	–	–	–
	**EORTC QLQ-C30**	PedsQL	30	0.64	(0.39, 0.90)	0.59	0.59	0.59
		PEDQOL	54	0.85	(0.77, 0.93)	0.80	0.80	0.80
**E3**	**PedsQL**	EORTC QLQ-C30	19	–	–	–	–	–
	**PEDQOL**		45	–	–	–	–	–
	**EORTC QLQ-C30**	PedsQL	19	0.87	(0.75, 0.99)	0.82	0.82	0.82
		PEDQOL	45	0.88	(0.81, 0.95)	0.83	0.83	0.83
**E4**	**PedsQL**	EORTC QLQ-C30	17	–	–	–	–	–
	**PEDQOL**		32	–	–	–	–	–
	**EORTC QLQ-C30**	PedsQL	17	0.73	(0.50, 0.97)	0.70	0.70	0.70
		PEDQOL	32	0.59	(0.35, 0.83)	0.59	0.59	0.59

**Table D.1:** Paediatric questionnaires and EORTC QLQ-C30: concordance measures.

Concordance Coefficient	Questionnaire	Domain					
		Functional				Symptom	
		Physical Functioning	Emotional Functioning	Cognitive Functioning	Social Functioning	Fatigue	Pain
Pearson's *r* (95% CI)	PedsQL PEDQOL	0.74 (0.64–0.82) 0.64 (0.54–0.72)	0.64 (0.52–0.74) 0.58 (0.47–0.67)	0.37 (0.17–0.54) 0.57 (0.45–0.67)	0.27 (0.07–0.44) 0.16 (−0.01–0.32)	0.70 (0.58–0.78) 0.72 (0.64–0.78)	0.59 (0.45–0.70) 0.73 (0.67–0.78)
Lin's *ρ* (95% CI)	PedsQL PEDQOL	0.74 (0.64–0.82) 0.64 (0.54–0.72)	0.55 (0.42–0.65) 0.36 (0.27–0.44)	0.37 (0.17-0-54) 0.47 (0.35–0.58)	0.17 (0.05–0.29) 0.08 (−0.01–0.16)	0.69 (0.58–0.78) 0.71 (0.63–0.77)	0.58 (0.45–0.69) 0.73 (0.65–0.79)

**Table E.1:** EURAMOS-1 consortium.

Name	Surname	Affiliation Dept/Programme/Centre	Institution Name	City	Country
Sigbjørn	Smeland	Institute for Clinical Medicine	Oslo University Hospital	Oslo	NO
Stefan S	Bielack	Olgahospital Stuttgart	Klinikum Stuttgart	Stuttgart	DE
Jeremy	Whelan		University College Hospital	London	UK
Mark	Bernstein	IWK Health Center	Dalhousie University	Halifax, NS	CA
Kirsten	Sundby Hall	Institute for Clinical Medicine	Oslo University Hospital	Oslo	NO
Catherine	Rechnitzer	Rigshospitalet	University of Copenhagen	Copenhagen	DK
Mikael	Eriksson		Lund University	Lund	SE
Imre	Antal		Semmelweis University	Budapest	HU
Godehard	Friedel	Thoracic surgery	Klinik Schillerhöhe	Gerlingen	DE
Stefanie	Hecker-Nolting	Olgahospital Stuttgart	Klinikum Stuttgart	Stuttgart	DE
Edita	Kabickova		Motol University Hospital	Prague	CZ
Leo	Kager		St. Anna Kinderspital/CCRI	Vienna	AT
Thomas	Kühne		University Hospital Basel	Basel	CH
Susanna	Lang		Medical University of Vienna	Vienna	AT
Regine	Mayer-Steinacker		University Hospital Ulm	Ulm	DE
Peter	Reichardt		HELIOS Klinikum Berlin-Buch	Berlin	DE
Beate	Timmermann		University Hospital Essen	Essen	DE
Thekla	von Kalle	Olgahospital Stuttgart	Klinikum Stuttgart	Stuttgart	DE
Carola	Arndt		Mayo Clinic	Rochester, MN	US
Ching C	Lau		Baylor College of Medicine	Houston, TX	US
Cindy L	Schwartz	M D Anderson Cancer Center	University of Texas	Houston, TX	US
Douglas S	Hawkins		University of Washington	Seattle, WA	US
Holcombe E	Grier		Dana-Farber Cancer Institute	Boston, MA	US
Katherine A	Janeway		Dana-Farber Cancer Institute	Boston, MA	US
Ken L B	Brown		University of British Columbia	Vancouver, BC	CA
Leo	Mascarenhas	Keck School of Medicine	University of Southern California	Los Angeles, CA	US
Lisa	Teot		Boston Children's Hospital	Boston, MA	US
Mark C	Gebhardt		Dana-Farber Cancer Institute	Boston, MA	US
Mark D	Krailo		Children's Oncology Group	Arcadia, CA	US
Michael S	Isakoff		Connecticut Children's Medical Center	Hartford, CT	US
Patrick J	Leavey	Southwestern Children's Medical Center	University of Texas	Dallas, TX	US
Paul A	Meyers		MSKCC	New York, NY	US
R Lor	Randall	Primary Childrens Hospital	The University of Utah	SLC, UT	US
Raj	Nagarajan		Children's Hospital Medical Center	Cincinnati, OH	US
Richard	Gorlick	M D Anderson Cancer Center	The University of Texas	Houston, TX	US
Robert	Goldsby	Paediatric Oncology	UCSF Medical Center–Mission Bay	SF, CA	US
Stephen L	Lessnick		Nationwide Children's Hospital/OSU	Columbus, OH	US
Catherina	Dhooge		University Hospital Ghent	Ghent	BE
Michael	Capra		Our Lady's Children's Hospital	Dublin	IE
Jakob	Anninga				NL
Adrienne M	Flanagan	Cancer Institute	RNOH/UCL	Stanmore/London	UK
Robert	Grimer		Royal Orthopaedic Hospital	Birmingham	UK
Sandra	Strauss		University College Hospital	London	UK
Hans	Gelderblom		Leiden University Medical Center	Leiden	NL
Marleen	Renard		University Hospital Leuven	Leuven	BE
Fiona	Ingleby	MRC Clinical Trials Unit	University College London	London	UK
Gordana	Jovic	MRC Clinical Trials Unit	University College London	London	UK
Trude	Butterfaß-Bahloul		University Hospital Münster	Münster	DE
Gabriele	Calaminus	Paediatric Haematology and Oncology	University Hospital Bonn	Bonn	DE
Pancras	Hogendoorn		Leiden University Medical Center	Leiden	NL
Matthew R	Sydes	MRC Clinical Trials Unit	University College London	London	UK
Neyssa	Marina		Five Prime Therapeutics, Inc	South SF, CA	US

**Table F.1:** PedsQL physical functioning items.

About My Health and Activities*(problems with …)*	Never	Almost Never	Sometimes	Often	Always
1. It is hard for me to walk more than one block.	□	□	□	□	□
2. It is hard for me to run.	□	□	□	□	□
3. It is hard for me to do sports activity or exercise.	□	□	□	□	□
4. It is hard for me to lift something heavy.	□	□	□	□	□
5. It is hard for me to take a bath or shower by myself.	□	□	□	□	□
6. It is hard for me to do chores around the house.	□	□	□	□	□
7. I hurt or ache.	□	□	□	□	□
8. I have low energy	□	□	□	□	□

**Table F.2:** PEDQOL physical functioning items.

In der letzten Woche …/In the last week …	Nie/Never	Selten/Rarely	Häufig/Frequently	Immer/Always
1…. konnte ich mit meinen Freunden beim Sport mithalten./… I was able to keep up with my friends in sports.	□	□	□	□
2…. habe ich beim Spielen und beim Sport lieber zugesehen als mitgespielt./ … I watched rather than played in games and sports.	□	□	□	□
3…. habe ich mich stark gefühlt./… I felt strong.	□	□	□	□
4…. fühlte ich mich fit genug, um nach der Schule mit meinen Freunden zu spielen/ … I felt fit enough to play with my friends after school.	□	□	□	□

**Table F.3:** EORTC QLQ-C30 physical functioning items.

DURING THE PAST WEEK:	Not at All	A Little	Quite a Bit	Very Much
1. Were you short of breath?	□	□	□	□
2. Have you had pain?	□	□	□	□
3. Did you need to rest?	□	□	□	□
4. Have you had trouble sleeping?	□	□	□	□
5. Have you felt weak?	□	□	□	□

**Table G.1:** PedsQL emotional functioning items.

About My Feelings*(problems with …)*	Never	Almost Never	Sometimes	Often	Always
1. I feel afraid or scared.	□	□	□	□	□
2. I feel sad or blue.	□	□	□	□	□
3. I feel angry.	□	□	□	□	□
4. I have trouble sleeping.	□	□	□	□	□
5. I worry about what will happen to me.	□	□	□	□	□

**Table G.2:** PEDQOL emotional functioning items.

In der letzten Woche …/In the last week …	Nie/Never	Selten/Rarely	Häufig/Frequently	Immer/Always
1…. fühlte ich mich alleine./ … I felt alone.	□	□	□	□
2…. war ich ärgerlich./ … I have been annoyed.	□	□	□	□
3…. fühlte ich mich glücklich./ … I felt happy.	□	□	□	□
4…. habe ich viel gelacht und Spaßgehabt./ … I have laughed a lot and had fun.	□	□	□	□

**Table G.3:** EORTC QLQ-C30 emotional functioning items.

DURING THE PAST WEEK:	Not at All	A Little	Quite a Bit	Very Much
1. Did you feel tense?	□	□	□	□
2. Did you worry?	□	□	□	□
3. Did you feel irritable?	□	□	□	□
4. Did you feel depressed?	□	□	□	□

**Table H.1:** PedsQL cognitive functioning items.

About School*(problems with …)*	Never	Almost Never	Sometimes	Often	Always
1. It is hard to pay attention in class.	□	□	□	□	□
2. I forget things.	□	□	□	□	□
3. I have trouble keeping up with my schoolwork.	□	□	□	□	□
4. I miss school because of not feeling well.	□	□	□	□	□
5. I miss school to go to the doctor or hospital.	□	□	□	□	□

**Table H.2:** PEDQOL cognitive functioning items.

In der letzten Woche …/In the last week …	Nie/Never	Selten/Rarely	Häufig/Frequently	Immer/Always
1…. fiel es mir leicht, neue Dinge zu lernen./ … I found it easy to learn new things.	□	□	□	□
2…. fiel es mir schwer, mich zu konzentrieren./ … I had a hard time concentrating.	□	□	□	□
3…. war ich genauso schlau wie alle anderen in der Klasse./ … I was just as smart as everyone else in the class.	□	□	□	□
4…. konnte ich mir Sachen gut merken./ … I've been able to remember things well.	□	□	□	□
5…. brauchte ich sehr lange, um meine Schularbeiten zu machen./ … it took me a long time to do my schoolwork.	□	□	□	□

**Table H.3:** EORTC QLQ-C30 cognitive functioning items.

DURING THE PAST WEEK:	Not at All	A Little	Quite a Bit	Very Much
1. Have you had difficulty concentrating on things, like reading a newspaper or watching television?	□	□	□	□
2. Have you had difficulty remembering things?	□	□	□	□

**Table I.1:** PedsQL social functioning items.

How I Get Along with Others (problems with …)	Never	Almost Never	Sometimes	Often	Always
1. I have trouble getting along with other kids.	□	□	□	□	□
2. Other kids do not want to be my friend.	□	□	□	□	□
3. Other kids tease me.	□	□	□	□	□
4. I cannot do things other kids my age can do.	□	□	□	□	□
5. It is hard to keep up when I play with other kids.	□	□	□	□	□

**Table I.2:** PEDQOL social functioning items.

In der letzten Woche …/In the last week …	Nie/Never	Selten/Rarely	Häufig/Frequently	Immer/Always
1… fühlte ich mich in Gruppen von Gleichaltrigen ausgeschlossen./ … I felt left out in groups of peers.	□	□	□	□
2… habe ich lieber was alleine gemacht./ … I preferred to do something on my own.	□	□	□	□
3… konnte ich mit meinen Freunden über das reden, was mir wirklich Sorgen macht./ … I was able to talk to my friends about what was really bothering me.	□	□	□	□
**Zum Schluss möchten wir Dich bitten, die folgenden allgemeinen Sätze zu beantworten:/Finally, we would like you to answer the following general sentences**:				
4. Ich habe es leicht, Freunde zu finden./I have an easy time making friends.	□	□	□	□
5. Ich bin beliebt bei meinen Freunden./I am popular with my friends.	□	□	□	□

**Table I.3:** EORTC QLQ-C30 social functioning items.

DURING THE PAST WEEK:	Not at All	A Little	Quite a Bit	Very Much
1. Has your physical condition or medical treatment interfered with your family life?	□	□	□	□
2. Has your physical condition or medical treatment interfered with your social activities?	□	□	□	□

**Table J.1:** PedsQL fatigue items.

About My Health and Activities*(problems with …)*	Never	Almost Never	Sometimes	Often	Always
1. I have low energy.	□	□	□	□	□

**Table J.2:** PEDQOL fatigue items.

In der letzten Woche …/In the last week …	Nie/Never	Selten/Rarely	Häufig/Frequently	Immer/Always
1…. fühlte ich mich schlapp und müde./ … I have felt listless and tired.	□	□	□	□

**Table J.3:** EORTC QLQ-C30 fatigue items.

DURING THE PAST WEEK:	Not at All	A Little	Quite a Bit	Very Much
1. Did you need to rest?	□	□	□	□
2. Have you felt weak?	□	□	□	□
3. Have you felt tired?	□	□	□	□

**Table K.1:** PedsQL pain items.

About My Health and Activities*(problems with …)*	Never	Almost Never	Sometimes	Often	Always
1. I hurt or ache.	□	□	□	□	□

**Table K.2:** PEDQOL pain items.

In der letzten Woche …/In the last week …	Nie/Never	Selten/Rarely	Häufig/Frequently	Immer/Always
1…. hatte ich Schmerzen./ … I've been in pain.	□	□	□	□

**Table K.3:** EORTC QLQ-C30 pain items.

DURING THE PAST WEEK:	Not at All	A Little	Quite a Bit	Very Much
1. Have you had pain?	□	□	□	□
2. Did pain interfere with your daily activities?	□	□	□	□

**Table L.1:** Crosswalk for *physical functioning.*

Original PedsQL score/PEDQOL score	Estimated EORTC QLQ-C30-equivalent score by linked questionnaire (Bootstrap *SE*)		Original PedsQL score/PEDQOL score	Estimated EORTC QLQ-C30-equivalent score by linked questionnaire (Bootstrap *SE*)	
	PedsQL	PEDQOL		PedsQL	PEDQOL
0	20 (5.45)	7 (1.76)	50	73 (2.79)	73 (3.11)
1	20 (5.69)	13 (2.79)	51	73 (2.72)	80 (3.06)
2	20 (5.62)	13 (3.30)	52	73 (2.64)	80 (3.01)
3	20 (5.48)	13 (3.60)	53	80 (2.57)	80 (2.95)
4	20 (5.30)	13 (3.78)	54	80 (2.49)	80 (2.90)
5	20 (5.10)	13 (3.89)	55	80 (2.41)	80 (2.84)
6	20 (4.89)	13 (3.94)	56	87 (2.34)	80 (2.78)
7	20 (4.68)	13 (3.95)	57	87 (2.26)	80 (2.72)
8	20 (4.47)	20 (3.93)	58	87 (2.19)	80 (2.66)
9	27 (4.27)	20 (3.89)	59	87 (2.12)	80 (2.60)
10	30 (4.95)	20 (3.66)	60	87 (2.05)	80 (2.54)
11	30 (4.74)	20 (3.60)	61	87 (1.98)	80 (2.47)
12	33 (4.55)	20 (3.53)	62	87 (1.91)	80 (2.41)
13	33 (4.37)	20 (3.47)	63	87 (1.85)	80 (2.35)
14	33 (4.21)	20 (3.41)	64	87 (1.78)	80 (2.28)
15	33 (4.06)	20 (3.36)	65	87 (1.72)	80 (2.22)
16	33 (3.92)	20 (3.31)	66	87 (1.65)	80 (2.16)
17	33 (3.80)	27 (3.27)	67	93 (1.59)	87 (2.09)
18	33 (3.69)	33 (3.25)	68	93 (1.53)	93 (2.03)
19	33 (3.60)	33 (3.22)	69	93 (1.46)	93 (1.96)
20	40 (3.52)	33 (3.21)	70	93 (1.40)	93 (1.89)
21	40 (3.45)	33 (3.20)	71	93 (1.34)	93 (1.83)
22	44 (3.40)	33 (3.20)	72	93 (1.28)	93 (1.77)
23	47 (3.35)	33 (3.20)	73	93 (1.22)	93 (1.70)
24	47 (3.31)	33 (3.20)	74	93 (1.16)	93 (1.64)
25	47 (3.28)	40 (3.21)	75	100 (1.11)	93 (1.58)
26	47 (3.25)	44 (3.22)	76	100 (1.05)	96 (1.51)
27	47 (3.24)	44 (3.23)	77	100 (1.00)	96 (1.45)
28	53 (3.23)	44 (3.25)	78	100 (0.95)	96 (1.39)
29	53 (3.22)	44 (3.26)	79	100 (0.90)	96 (1.34)
30	53 (3.22)	44 (3.28)	80	100 (0.85)	96 (1.28)
31	53 (3.22)	44 (3.29)	81	100 (0.80)	96 (1.22)
32	56 (3.22)	44 (3.30)	82	100 (0.76)	96 (1.16)
33	56 (3.22)	47 (3.32)	83	100 (0.71)	100 (1.11)
34	60 (3.23)	53 (3.33)	84	100 (0.67)	100 (1.05)
35	60 (3.24)	53 (3.34)	85	100 (0.63)	100 (1.00)
36	60 (3.24)	53 (3.35)	86	100 (0.59)	100 (0.95)
37	60 (3.24)	53 (3.36)	87	100 (0.55)	100 (0.89)
38	60 (3.24)	53 (3.36)	88	100 (0.51)	100 (0.84)
39	64 (3.24)	53 (3.36)	89	100 (0.47)	100 (0.78)
40	64 (3.23)	53 (3.36)	90	100 (0.43)	100 (0.73)
41	70 (3.22)	53 (3.35)	91	100 (0.40)	100 (0.67)
42	73 (3.20)	60 (3.35)	92	100 (0.36)	100 (0.61)
43	73 (3.17)	67 (3.33)	93	100 (0.32)	100 (0.55)
44	73 (3.14)	67 (3.31)	94	100 (0.28)	100 (0.49)
45	73 (3.09)	67 (3.29)	95	100 (0.24)	100 (0.42)
46	73 (3.05)	67 (3.26)	96	100 (0.20)	100 (0.35)
47	73 (2.99)	67 (3.23)	97	100 (0.16)	100 (0.28)
48	73 (2.93)	67 (3.19)	98	100 (0.12)	100 (0.21)
49	73 (2.86)	67 (3.15)	99	100 (0.07)	100 (0.13)
			100	100 (0.02)	100 (0.04)

**Table L.2:** Crosswalk for *emotional functioning.*

Original PedsQL score/PEDQOL score	Estimated EORTC QLQ-C30-equivalent score by linked questionnaire (Bootstrap *SE*)		Original PedsQL score/PEDQOL score	Estimated EORTC QLQ-C30-equivalent score by linked questionnaire (Bootstrap *SE*)	
	PedsQL	PEDQOL		PedsQL	PEDQOL
0	0 (4.04)	0 (1.33)	50	58 (4.47)	42 (2.82)
1	0 (4.74)	0 (1.94)	51	62 (4.62)	42 (2.79)
2	0 (5.16)	0 (2.32)	52	62 (4.76)	42 (2.77)
3	0 (5.48)	0 (2.61)	53	62 (4.86)	42 (2.75)
4	0 (5.75)	0 (2.85)	54	62 (4.92)	42 (2.73)
5	0 (5.99)	0 (3.07)	55	67 (4.92)	42 (2.72)
6	0 (6.21)	0 (3.28)	56	67 (4.86)	42 (2.71)
7	0 (6.41)	0 (3.47)	57	67 (4.74)	42 (2.71)
8	0 (6.59)	0 (3.65)	58	67 (4.58)	50 (2.71)
9	0 (6.75)	0 (3.82)	59	67 (4.38)	58 (2.72)
10	0 (6.90)	0 (3.99)	60	75 (4.15)	58 (2.72)
11	0 (7.02)	0 (4.16)	61	83 (3.91)	58 (2.74)
12	0 (7.13)	0 (4.33)	62	83 (3.67)	58 (2.76)
13	0 (7.21)	0 (4.49)	63	83 (3.43)	58 (2.78)
14	0 (7.26)	0 (4.64)	64	83 (3.21)	58 (2.80)
15	17 (7.29)	0 (4.79)	65	83 (2.99)	58 (2.82)
16	17 (7.28)	0 (4.93)	66	83 (2.80)	58 (2.84)
17	17 (7.24)	0 (5.07)	67	83 (2.62)	58 (2.86)
18	17 (7.17)	16 (5.19)	68	83 (2.45)	67 (2.87)
19	17 (7.06)	16 (5.30)	69	83 (2.31)	67 (2.87)
20	21 (6.93)	16 (5.39)	70	83 (2.17)	67 (2.86)
21	29 (6.76)	16 (5.47)	71	92 (2.05)	67 (2.83)
22	29 (6.56)	16 (5.53)	72	92 (1.93)	67 (2.79)
23	29 (6.34)	16 (5.57)	73	92 (1.83)	67 (2.73)
24	29 (6.10)	16 (5.59)	74	92 (1.74)	67 (2.66)
25	29 (5.84)	17 (5.59)	75	92 (1.65)	83 (2.58)
26	29 (5.58)	17 (5.56)	76	92 (1.56)	83 (2.48)
27	29 (5.31)	17 (5.51)	77	92 (1.49)	83 (2.38)
28	29 (5.05)	17 (5.44)	78	92 (1.41)	83 (2.28)
29	29 (4.80)	17 (5.35)	79	92 (1.34)	83 (2.17)
30	33 (4.56)	17 (5.23)	80	100 (1.28)	83 (2.06)
31	33 (4.35)	17 (5.10)	81	100 (1.22)	83 (1.96)
32	33 (4.15)	17 (4.95)	82	100 (1.16)	83 (1.85)
33	33 (3.98)	17 (4.79)	83	100 (1.10)	92 (1.75)
34	33 (3.84)	21 (4.62)	84	100 (1.04)	92 (1.65)
35	33 (3.72)	21 (4.45)	85	100 (0.99)	92 (1.56)
36	33 (3.63)	21 (4.27)	86	100 (0.94)	92 (1.47)
37	33 (3.55)	21 (4.10)	87	100 (0.89)	92 (1.37)
38	33 (3.51)	21 (3.93)	88	100 (0.84)	92 (1.28)
39	33 (3.48)	21 (3.78)	89	100 (0.79)	92 (1.19)
40	42 (3.47)	21 (3.63)	90	100 (0.74)	92 (1.10)
41	50 (3.49)	21 (3.49)	91	100 (0.69)	92 (1.01)
42	50 (3.52)	33 (3.37)	92	100 (0.64)	100 (0.92)
43	50 (3.57)	33 (3.26)	93	100 (0.59)	100 (0.82)
44	50 (3.65)	33 (3.17)	94	100 (0.54)	100 (0.73)
45	50 (3.74)	33 (3.08)	95	100 (0.48)	100 (0.63)
46	58 (3.86)	33 (3.01)	96	100 (0.41)	100 (0.52)
47	58 (3.99)	33 (2.95)	97	100 (0.34)	100 (0.42)
48	58 (4.14)	33 (2.90)	98	100 (0.25)	100 (0.30)
49	58 (4.30)	33 (2.86)	99	100 (0.16)	100 (0.18)
			100	100 (0.06)	100 (0.06)

**Table L.3:** Crosswalk for *cognitive functioning.*

Original PedsQL score/PEDQOL score	Estimated EORTC QLQ-C30-equivalent score by linked questionnaire (Bootstrap *SE*)		Original PedsQL score/PEDQOL score	Estimated EORTC QLQ-C30-equivalent score by linked questionnaire (Bootstrap *SE*)	
	PedsQL	PEDQOL		PedsQL	PEDQOL
0	0 (10.88)	0 (5.96)	50	83 (5.84)	67 (5.60)
1	0 (11.03)	16 (6.55)	51	83 (5.40)	67 (5.73)
2	0 (11.02)	16 (6.77)	52	83 (4.96)	67 (5.83)
3	0 (10.96)	16 (6.87)	53	83 (4.54)	67 (5.88)
4	0 (10.87)	16 (6.91)	54	83 (4.13)	67 (5.88)
5	0 (10.75)	16 (6.90)	55	83 (3.76)	67 (5.82)
6	0 (10.60)	16 (6.86)	56	83 (3.42)	67 (5.70)
7	0 (10.42)	17 (6.80)	57	83 (3.11)	67 (5.50)
8	0 (10.22)	17 (6.71)	58	83 (2.85)	67 (5.25)
9	0 (9.99)	17 (6.61)	59	83 (2.61)	67 (4.94)
10	0 (9.75)	17 (6.49)	60	83 (2.40)	67 (4.60)
11	0 (9.47)	17 (6.37)	61	83 (2.22)	83 (4.25)
12	0 (9.18)	17 (6.23)	62	83 (2.07)	83 (3.88)
13	0 (8.86)	17 (6.09)	63	83 (1.93)	83 (3.53)
14	0 (8.54)	17 (5.95)	64	83 (1.81)	83 (3.19)
15	0 (8.22)	17 (5.80)	65	83 (1.71)	83 (2.88)
16	0 (7.89)	17 (5.66)	66	83 (1.61)	83 (2.60
17	0 (7.54)	17 (5.51)	67	83 (1.53)	83 (2.34)
18	0 (7.19)	17 (5.37)	68	83 (1.45)	83 (2.12)
19	0 (6.85)	17 (5.23)	69	83 (1.39)	83 (1.92)
20	0 (6.53)	17 (5.09)	70	83 (1.32)	83 (1.75)
21	0 (6.23)	25 (4.96)	71	83 (1.26)	83 (1.59)
22	0 (5.97)	25 (4.84)	72	83 (1.21)	83 (1.46)
23	0 (5.72)	25 (4.72)	73	83 (1.16)	100 (1.34)
24	0 (5.51)	25 (4.62)	74	83 (1.11)	100 (1.23)
25	42 (5.33)	25 (4.52)	75	100 (1.07)	100 (1.13)
26	50 (5.16)	25 (4.44)	76	100 (1.03)	100 (1.05)
27	50 (5.03)	33 (4.36)	77	100 (0.99)	100 (0.97)
28	50 (4.92)	33 (4.30)	78	100 (0.95)	100 (0.89)
29	50 (4.84)	33 (4.24)	79	100 (0.91)	100 (0.83)
30	50 (4.78)	33 (4.20)	80	100 (0.87)	100 (0.77)
31	50 (4.76)	33 (4.17)	81	100 (0.84)	100 (0.71)
32	50 (4.77)	33 (4.15)	82	100 (0.80)	100 (0.66)
33	50 (4.81)	33 (4.14)	783	100 (0.77)	100 (0.61)
34	50 (4.90)	42 (4.14)	84	100 (0.73)	100 (0.56)
35	50 (5.03)	42 (4.15)	85	100 (0.70)	100 (0.52)
36	50 (5.20)	42 (4.17)	86	100 (0.67)	100 (0.48)
37	50 (5.42)	42 (4.20)	87	100 (0.64)	100 (0.44)
38	67 (5.68)	42 (4.24)	88	100 (0.60)	100 (0.40)
39	67 (5.96)	42 (4.30)	89	100 (0.57)	100 (0.37)
40	67 (6.26)	50 (4.36)	90	100 (0.54)	100 (0.34)
41	67 (6.56)	50 (4.44)	91	100 (0.50)	100 (0.31)
42	67 (6.82)	50 (4.53)	92	100 (0.47)	100 (0.28)
43	67 (7.03)	50 (4.63)	93	100 (0.44)	100 (0.24)
44	67 (7.15)	50 (4.74)	94	100 (0.40)	100 (0.21)
45	67 (7.17)	50 (4.87)	95	100 (0.35)	100 (0.18)
46	67 (7.08)	50 (5.00)	96	100 (0.30)	100 (0.15)
47	67 (6.88)	67 (5.15)	97	100 (0.25)	100 (0.12)
48	67 (6.60)	67 (5.30)	98	100 (0.19)	100 (0.09)
49	67 (6.24)	67 (5.46)	99	100 (0.12)	100 (0.06)
			100	100 (0.05)	100 (0.02)

**Table L.4:** Crosswalk for *social functioning.*

Original PedsQL score/PEDQOL score	Estimated EORTC QLQ-C30-equivalent score by linked questionnaire (Bootstrap *SE*)		Original PedsQL score/PEDQOL score	Estimated EORTC QLQ-C30-equivalent score by linked questionnaire (Bootstrap *SE*)	
	PedsQL	PEDQOL		PedsQL	PEDQOL
0	0 (0.1)	0 (8e-04)	50	0 (4.5)	0 (1e+00)
1	0 (0.2)	0 (2e-03)	51	0 (4.6)	0 (1e+00)
2	0 (0.2)	0 (3e-03)	52	0 (4.7)	0 (2e+00)
3	0 (0.3)	0 (4e-03)	53	0 (4.7)	0 (2e+00)
4	0 (0.3)	0 (4e-03)	54	0 (4.7)	0 (2e+00)
5	0 (0.3)	0 (5e-03)	55	17 (4.7)	0 (2e+00)
6	0 (0.3)	0 (5e-03)	56	17 (4.7)	0 (2e+00)
7	0 (0.3)	0 (6e-03)	57	17 (4.6)	0 (3e+00)
8	0 (0.3)	0 (6e-03)	58	17 (4.6)	0 (3e+00)
9	0 (0.4)	0 (6e-03)	59	17 (4.5)	0 (3e+00)
10	0 (0.4)	0 (7e-03)	60	33 (4.5)	0 (3e+00)
11	0 (0.4)	0 (7e-03)	61	33 (4.5)	0 (3e+00)
12	0 (0.4)	0 (7e-03)	62	33 (4.5)	0 (3e+00)
13	0 (0.4)	0 (8e-03)	63	33 (4.5)	0 (4e+00)
14	0 (0.4)	0 (8e-03)	64	33 (4.6)	0 (4e+00)
15	0 (0.4)	0 (9e-03)	65	33 (4.7)	0 (4e+00)
16	0 (0.4)	0 (9e-03)	66	33 (4.8)	0 (4e+00)
17	0 (0.4)	0 (1e-02)	67	33 (4.9)	17 (4e+00)
18	0 (0.4)	0 (1e-02)	68	33 (5.1)	17 (5e+00)
19	0 (0.5)	0 (1e-02)	69	33 (5.4)	17 (5e+00)
20	0 (0.5)	0 (1e-02)	70	50 (5.7)	17 (5e+00)
21	0 (0.5)	0 (1e-02)	71	50 (6.0)	17 (5e+00)
22	0 (0.5)	0 (1e-02)	72	50 (6.3)	17 (5e+00)
23	0 (0.5)	0 (2e-02)	73	50 (6.7)	33 (6e+00)
24	0 (0.6)	0 (2e-02)	74	50 (7.1)	50 (6e+00)
25	0 (0.6)	0 (2e-02)	75	50 (7.4)	50 (6e+00)
26	0 (0.7)	0 (2e-02)	76	67 (7.7)	50 (6e+00)
27	0 (0.7)	0 (3e-02)	77	67 (7.8)	50 (7e+00)
28	0 (0.8)	0 (3e-02)	78	67 (7.8)	50 (7e+00)
29	0 (0.8)	0 (4e-02)	79	67 (7.6)	50 (7e+00)
30	0 (0.9)	0 (4e-02)	80	67 (7.3)	50 (7e+00)
31	0 (1.0)	0 (5e-02)	81	75 (6.9)	67 (7e+00)
32	0 (1.1)	0 (6e-02)	82	75 (6.3)	67 (8e+00)
33	0 (1.2)	0 (8e-02)	83	75 (5.7)	67 (8e+00)
34	0 (1.3)	0 (9e-02)	84	75 (5.0)	67 (8e+00)
35	0 (1.5)	0 (1e-01)	85	83 (4.4)	67 (8e+00)
36	0 (1.6)	0 (1e-01)	86	100 (3.8)	67 (8e+00)
37	0 (1.8)	0 (2e-01)	87	100 (3.3)	67 (8e+00)
38	0 (2.0)	0 (2e-01)	88	100 (2.8)	83 (7e+00)
39	0 (2.2)	0 (2e-01)	89	100 (2.5)	83 (7e+00)
40	0 (2.4)	0 (3e-01)	90	100 (2.1)	83 (7e+00)
41	0 (2.6)	0 (3e-01)	91	100 (1.8)	83 (6e+00)
42	0 (2.9)	0 (4e-01)	92	100 (1.6)	83 (6e+00)
43	0 (3.1)	0 (5e-01)	93	100 (1.4)	83 (5e+00)
44	0 (3.4)	0 (6e-01)	94	100 (1.2)	83 (4e+00)
45	0 (3.6)	0 (7e-01)	95	100 (1.0)	83 (4e+00)
46	0 (3.8)	0 (8e-01)	96	100 (0.8)	83 (3e+00)
47	0 (4.1)	0 (9e-01)	97	100 (0.6)	83 (3e+00)
48	0 (4.2)	0 (1e+00)	98	100 (0.5)	83 (2e+00)
49	0 (4.4)	0 (1e+00)	99	100 (0.3)	83 (1e+00)
			100	100 (0.1)	100 (4e-01)

**Table L.5:** Crosswalk for *fatigue.*

Original PedsQL score/PEDQOL score	Estimated EORTC QLQ-C30-equivalent score by linked questionnaire (Bootstrap *SE*)		Original PedsQL score/PEDQOL score	Estimated EORTC QLQ-C30-equivalent score by linked questionnaire (Bootstrap *SE*)	
	PedsQL	PEDQOL		PedsQL	PEDQOL
0	0 (0.2)	11 (0.7)	50	33 (4.0)	56 (2.7)
1	10 (0.4)	22 (1.4)	51	56 (4.1)	56 (2.7)
2	10 (0.7)	22 (1.8)	52	56 (4.1)	56 (2.8)
3	10 (0.9)	22 (2.0)	53	56 (4.1)	56 (2.8)
4	10 (1.1)	22 (2.1)	54	56 (4.1)	56 (2.8)
5	10 (1.2)	22 (2.2)	55	56 (4.1)	56 (2.9)
6	10 (1.3)	22 (2.2)	56	56 (4.1)	56 (2.9)
7	10 (1.5)	22 (2.2)	57	56 (4.1)	56 (2.9)
8	10 (1.6)	22 (2.2)	58	56 (4.1)	56 (3.0)
9	10 (1.7)	22 (2.2)	59	56 (4.1)	56 (3.0)
10	10 (1.8)	22 (2.3)	60	56 (4.1)	56 (3.0)
11	10 (1.9)	22 (2.3)	61	56 (4.0)	56 (3.1)
12	10 (1.9)	22 (2.3)	62	56 (4.0)	56 (3.1)
13	10 (2.0)	22 (2.3)	63	56 (4.0)	56 (3.1)
14	10 (2.1)	22 (2.3)	64	56 (4.0)	56 (3.1)
15	10 (2.2)	22 (2.3)	65	56 (4.0)	56 (3.2)
16	10 (2.2)	22 (2.3)	66	56 (3.9)	56 (3.2)
17	10 (2.3)	22 (2.3)	67	56 (3.9)	67 (3.2)
18	10 (2.4)	22 (2.3)	68	56 (3.9)	94 (3.2)
19	10 (2.4)	22 (2.3)	69	56 (3.9)	94 (3.2)
20	10 (2.5)	22 (2.3)	70	56 (3.9)	94 (3.2)
21	10 (2.6)	22 (2.3)	71	56 (3.8)	94 (3.2)
22	10 (2.6)	22 (2.3)	72	56 (3.8)	94 (3.2)
23	10 (2.7)	22 (2.4)	73	56 (3.8)	94 (3.2)
24	10 (2.8)	22 (2.4)	74	56 (3.8)	94 (3.2)
25	11 (2.8)	22 (2.4)	75	67 (3.8)	94 (3.1)
26	22 (2.9)	22 (2.4)	76	78 (3.8)	94 (3.1)
27	22 (3.0)	22 (2.4)	77	78 (3.8)	94 (3.1)
28	22 (3.1)	22 (2.4)	78	78 (3.9)	94 (3.1)
29	22 (3.1)	22 (2.4)	79	78 (3.9)	94 (3.0)
30	22 (3.2)	22 (2.4)	80	78 (3.9)	94 (3.0)
31	22 (3.3)	22 (2.4)	81	78 (4.0)	94 (2.9)
32	22 (3.3)	22 (2.4)	82	78 (4.0)	94 (2.8)
33	22 (3.4)	33 (2.5)	83	78 (4.1)	94 (2.8)
34	22 (3.4)	56 (2.5)	84	78 (4.2)	94 (2.7)
35	22 (3.5)	56 (2.5)	85	78 (4.3)	94 (2.6)
36	22 (3.6)	56 (2.5)	86	78 (4.3)	94 (2.4)
37	22 (3.6)	56 (2.5)	87	78 (4.4)	94 (2.3)
38	22 (3.7)	56 (2.5)	88	78 (4.5)	94 (2.2)
39	22 (3.7)	56 (2.5)	89	78 (4.6)	94 (2.0)
40	22 (3.8)	56 (2.5)	90	78 (4.6)	94 (1.9)
41	22 (3.8)	56 (2.5)	91	78 (4.7)	94 (1.7)
42	22 (3.8)	56 (2.5)	92	78 (4.7)	94 (1.5)
43	22 (3.9)	56 (2.5)	93	78 (4.6)	94 (1.4)
44	22 (3.9)	56 (2.5)	94	78 (4.6)	94 (1.2)
45	22 (3.9)	56 (2.6)	95	78 (4.5)	94 (1.0)
46	22 (4.0)	56 (2.6)	96	78 (4.3)	94 (0.8)
47	22 (4.0)	56 (2.6)	97	78 (4.1)	94 (0.7)
48	22 (4.0)	56 (2.6)	98	78 (3.7)	94 (0.5)
49	22 (4.0)	56 (2.7)	99	78 (3.1)	94 (0.3)
			100	89 (2.1)	100 (0.1)

**Table L.6:** Crosswalk for *pain.*

Original PedsQL score/PEDQOL score	Estimated EORTC QLQ-C30-equivalent score by linked questionnaire (Bootstrap *SE*)		Original PedsQL score/PEDQOL score	Estimated EORTC QLQ-C30-equivalent score by linked questionnaire (Bootstrap *SE*)	
	PedsQL	PEDQOL		PedsQL	PEDQOL
0	0 (0.09)	0 (0.07)	50	33 (5.90)	50 (5.84)
1	0 (0.24)	0 (0.20)	51	67 (6.06)	50 (5.94)
2	0 (0.39)	0 (0.32)	52	67 (6.21)	50 (6.01)
3	0 (0.52)	0 (0.42)	53	67 (6.34)	50 (6.04)
4	0 (0.64)	0 (0.52)	54	67 (6.46)	50 (6.04)
5	0 (0.74)	0 (0.60)	55	67 (6.57)	50 (5.99)
6	0 (0.84)	0 (0.68)	56	67 (6.65)	50 (5.92)
7	0 (0.93)	0 (0.76)	57	67 (6.72)	50 (5.81)
8	0 (1.02)	0 (0.83)	58	67 (6.76)	50 (5.68)
9	0 (1.10)	0 (0.89)	59	67 (6.78)	50 (5.53)
10	0 (1.17)	0 (0.96)	60	67 (6.78)	50 (5.37)
11	0 (1.25)	0 (1.02)	61	67 (6.76)	50 (5.20)
12	0 (1.32)	0 (1.08)	62	67 (6.72)	50 (5.02)
13	0 (1.39)	0 (1.14)	63	67 (6.67)	50 (4.86)
14	0 (1.46)	0 (1.21)	64	67 (6.60)	50 (4.69)
15	0 (1.53)	0 (1.27)	65	67 (6.52)	50 (4.53)
16	0 (1.61)	0 (1.34)	66	67 (6.44)	50 (4.39)
17	0 (1.68)	0 (1.40)	67	67 (6.35)	67 (4.25)
18	0 (1.75)	0 (1.47)	68	67 (6.26)	100 (4.12)
19	0 (1.83)	0 (1.54)	69	67 (6.16)	100 (4.00)
20	0 (1.91)	0 (1.62)	70	67 (6.07)	100 (3.89)
21	0 (1.99)	0 (1.70)	71	67 (5.98)	100 (3.80)
22	0 (2.07)	0 (1.77)	72	67 (5.89)	100 (3.72)
23	0 (2.16)	0 (1.86)	73	67 (5.81)	100 (3.65)
24	0 (2.25)	0 (1.94)	74	67 (5.74)	100 (3.59)
25	17 (2.34)	0 (2.03)	75	67 (5.66)	100 (3.53)
26	17 (2.44)	0 (2.13)	76	92 (5.59)	100 (3.49)
27	17 (2.54)	0 (2.22)	77	92 (5.52)	100 (3.45)
28	17 (2.64)	0 (2.33)	78	92 (5.46)	100 (3.41)
29	17 (2.75)	0 (2.43)	79	92 (5.39)	100 (3.37)
30	17 (2.86)	0 (2.54)	80	92 (5.32)	100 (3.33)
31	17 (2.98)	0 (2.66)	81	92 (5.24)	100 (3.28)
32	17 (3.10)	0 (2.78)	82	92 (5.16)	100 (3.23)
33	17 (3.22)	17 (2.91)	83	92 (5.07)	100 (3.16)
34	17 (3.35)	50 (3.05)	84	92 (4.97)	100 (3.08)
35	17 (3.49)	50 (3.19)	85	92 (4.87)	100 (3.00)
36	17 (3.63)	50 (3.34)	86	92 (4.75)	100 (2.89)
37	17 (3.77)	50 (3.50)	87	92 (4.61)	100 (2.78)
38	17 (3.92)	50 (3.67)	88	92 (4.47)	100 (2.65)
39	17 (4.07)	50 (3.84)	89	92 (4.30)	100 (2.51)
40	17 (4.22)	50 (4.02)	90	92 (4.12)	100 (2.36)
41	17 (4.38)	50 (4.21)	91	92 (3.93)	100 (2.19)
42	17 (4.55)	50 (4.40)	92	92 (3.71)	100 (2.01)
43	17 (4.71)	50 (4.60)	93	92 (3.47)	100 (1.83)
44	17 (4.88)	50 (4.80)	94	92 (3.21)	100 (1.63)
45	17 (5.05)	50 (5.00)	95	92 (2.92)	100 (1.42)
46	17 (5.22)	50 (5.20)	96	92 (2.61)	100 (1.21)
47	17 (5.40)	50 (5.38)	97	92 (2.25)	100 (0.98)
48	17 (5.57)	50 (5.56)	98	92 (1.83)	100 (0.74)
49	17 (5.74)	50 (5.71)	99	92 (1.34)	100 (0.48)
			100	100 (0.66)	100 (0.18)

^2^Score differences are defined as the paediatric instrument as less than the adult instrument.

^3^The US-English versions of are displayed here in an exemplary fashion.

## A Software

We conducted all statistical analyses using version 4.1.0 of the *R* platform, version 2.0.7 of *R* package *equate* for score linking, *R* package *blandr* for the calculation of concordance correlation coefficients and the *tidyverse* suite of *R* packages for data preparation and data visualisation.

## F. Physical *functioning* items per questionnaire.

### F.1 PedsQL physical functioning items.

*In the past* ***ONE month,*** *how much of a* ***problem*** *has this been for you …*

## G. *Emotional functioning* items per questionnaire.

### G.1 PedsQL emotional functioning items.

*In the past* ***ONE month,*** *how much of a* ***problem*** *has this been for you …*

## H. Cognitive functioning items per questionnaire.

### H.1 PedsQL cognitive functioning items.

*In the past* ***ONE month,*** *how much of a* ***problem*** *has this been for you …*

## I. Social functioning items per questionnaire.

### I.1 PedsQL social functioning items.

*In the past* ***ONE month,*** *how much of a* ***problem*** *has this been for you …*

## J. *Fatigue* items per questionnaire.

### J.1 PedsQL fatigue items.

*In the past* ***ONE month,*** *how much of a* ***problem*** *has this been for you …*

## K. *Pain* items per questionnaire.

### K.1 PedsQL pain items.

*In the past* ***ONE month,*** *how much of a* ***problem*** *has this been for you …*
