# Erratum to ‘Linking EORTC QLQ-C-30 and PedsQL/PEDQOL physical functioning scores in patients with osteosarcoma’ [Eur J Cancer 170 (2022) 209-235]

**DOI:** 10.1016/j.ejca.2022.06.053

**Published:** 2022-12

**Authors:** Axel Budde, Katja Baust, Leonie Weinhold, Mark Bernstein, Stefan Bielack, Catharina Dhooge, Lars Hjorth, Katherine A. Janeway, Meriel Jenney, Mark D. Krailo, Neyssa Marina, Rajaram Nagarajan, Sigbjørn Smeland, Matthew R. Sydes, Patricia De Vos, Jeremy Whelan, Andreas Wiener, Gabriele Calaminus, Matthias Schmid

**Affiliations:** a Department of Paediatric Haematology and Oncology, University Hospital Bonn, Bonn, Germany; b Department of Medical Biometry, Informatics, and Epidemiology, University Hospital Bonn, Bonn, Germany; c IWK Health Centre, Dalhousie University, Halifax, NS, Canada; d Zentrum fu¨r Kinder-, Jugend- und Frauenmedizin, Pa¨diatrie, Klinikum Stuttgart, Olgahospital, Stuttgart, Germany; e Department of Internal Medicine and Paediatrics, Faculty of Medicine and Health Sciences, Ghent University Hospital, Ghent, Belgium; f Department of Clinical Sciences, Department of Paediatrics, Lund University, Skane University Hospital, Lund, Sweden; g Dana-Farber/Boston Children’s Cancer and Blood Disorders Center, Harvard Medical School, Boston, MA, USA; h Women’s Services Clinical Board, University Hospital of Wales, Cardiff, UK; i Statistics and Data Center, Children’s Oncology Group, Monrovia, CA, USA; j Five Prime Therapeutics, South San Francisco, CA, USA; k Division of Oncology, Cincinnati Children’s Hospital Medical Center, University of Cincinnati College of Medicine, Cincinnati, OH, USA; l Norwegian Radium Hospital, Oslo University Hospital, Oslo, Norway; m MRC Clinical Trials Unit at UCL, Institute of Clinical Trials and Methodology, University College London, London, UK; n Department of Paediatric Haematology and Oncology, Ghent University Hospital, Ghent, Belgium; o Department of Oncology, University College Hospital, London, UK; p West German Proton Therapy Center Essen, Essen, Germany

The publisher regrets that errors appear in Table 2a in the printed article. These errors have now been corrected online, and the correct version of Table 2a is given here.

**Figure:**
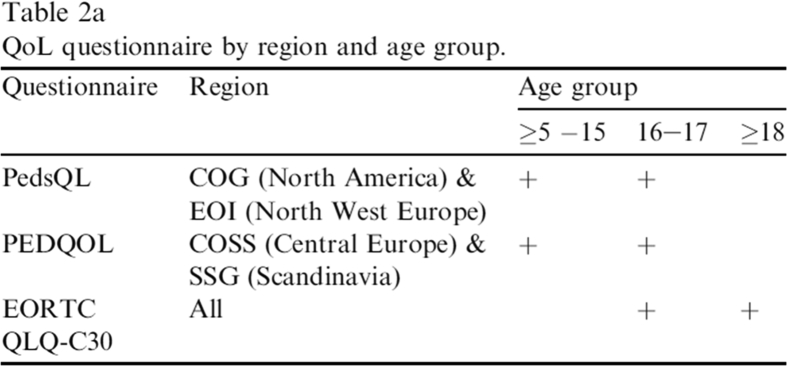

We also note that a Multimedia component was included with the online article in error. This has now been removed from the online article. We can confirm that there are no additional Supplementary Data files to appear with this article.

The publisher would like to apologise for any inconvenience caused.

