# Supplementary figures and images for: Linking EORTC QLQ-C-30 and PedsQL/PEDQOL physical functioning scores in patients with osteosarcoma
Source: Eur J Cancer. 2022 Jul;170:209–35. doi: 10.1016/j.ejca.2022.03.018 (PMC9251607; doi:10.1016/j.ejca.2022.03.018)

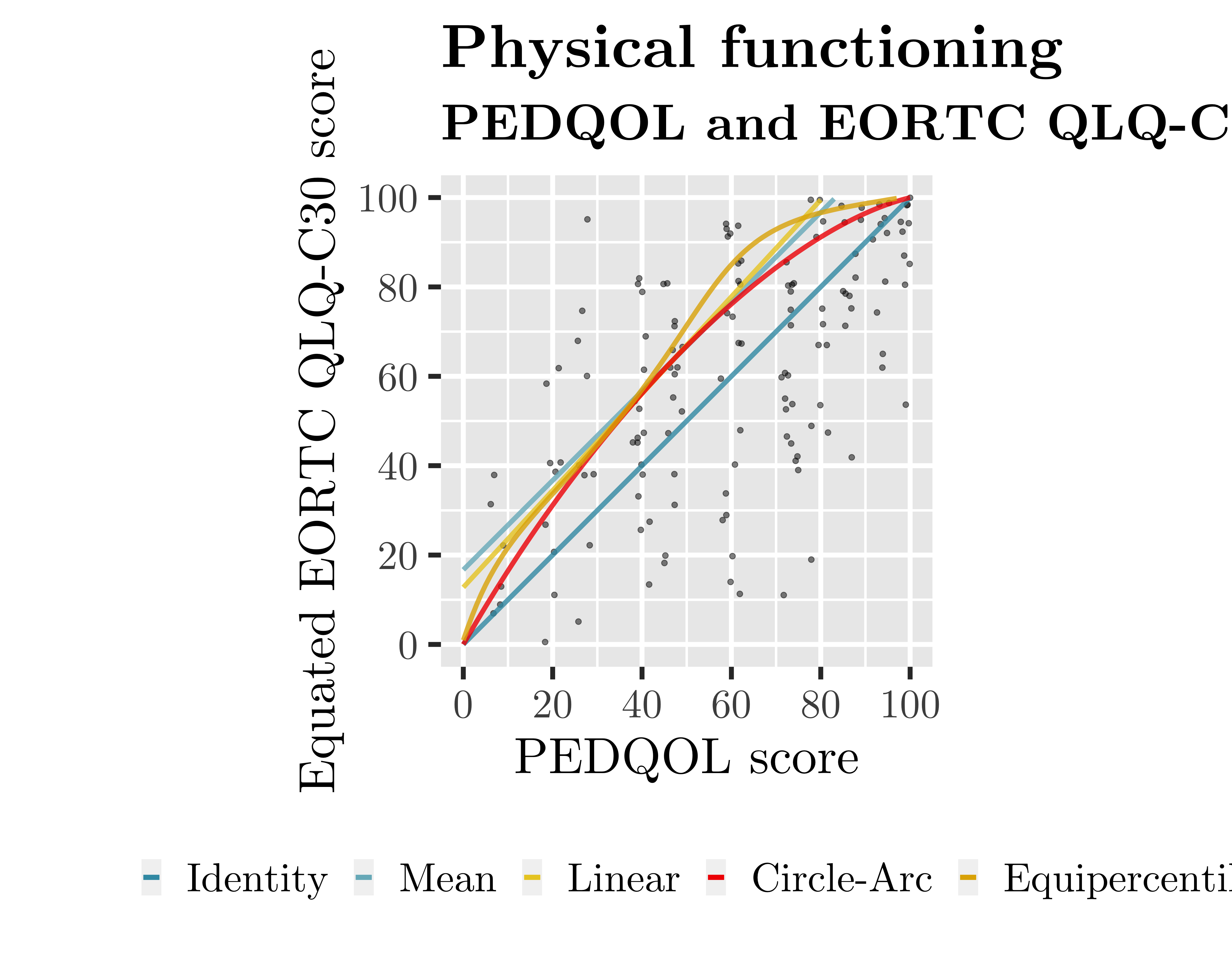

Supplement: Multimedia component 1 [file mmc1.zip › pedqol_eortc_functions_physical.png]

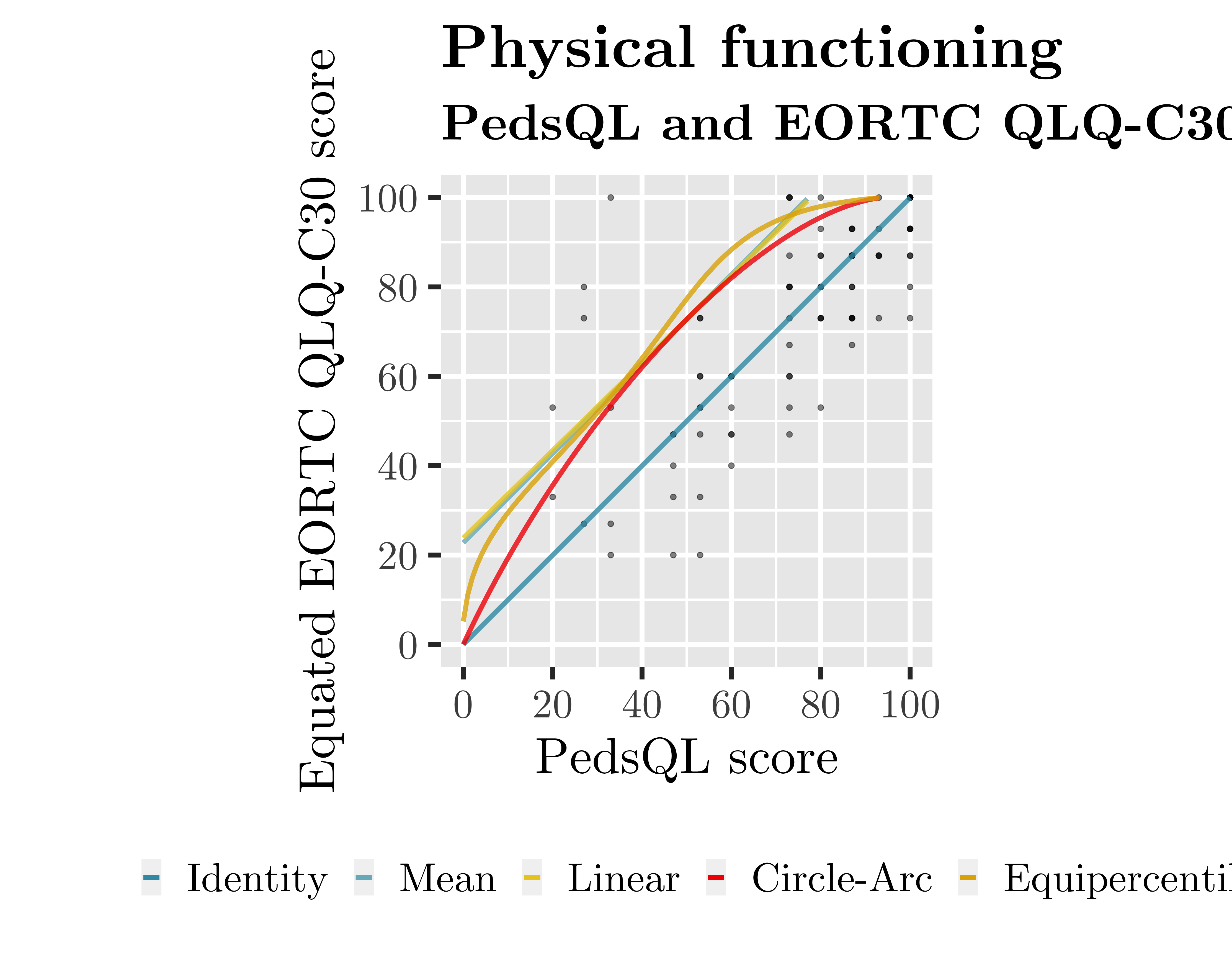

Supplement: Multimedia component 1 [file mmc1.zip › pedsql_eortc_functions_physical.png]

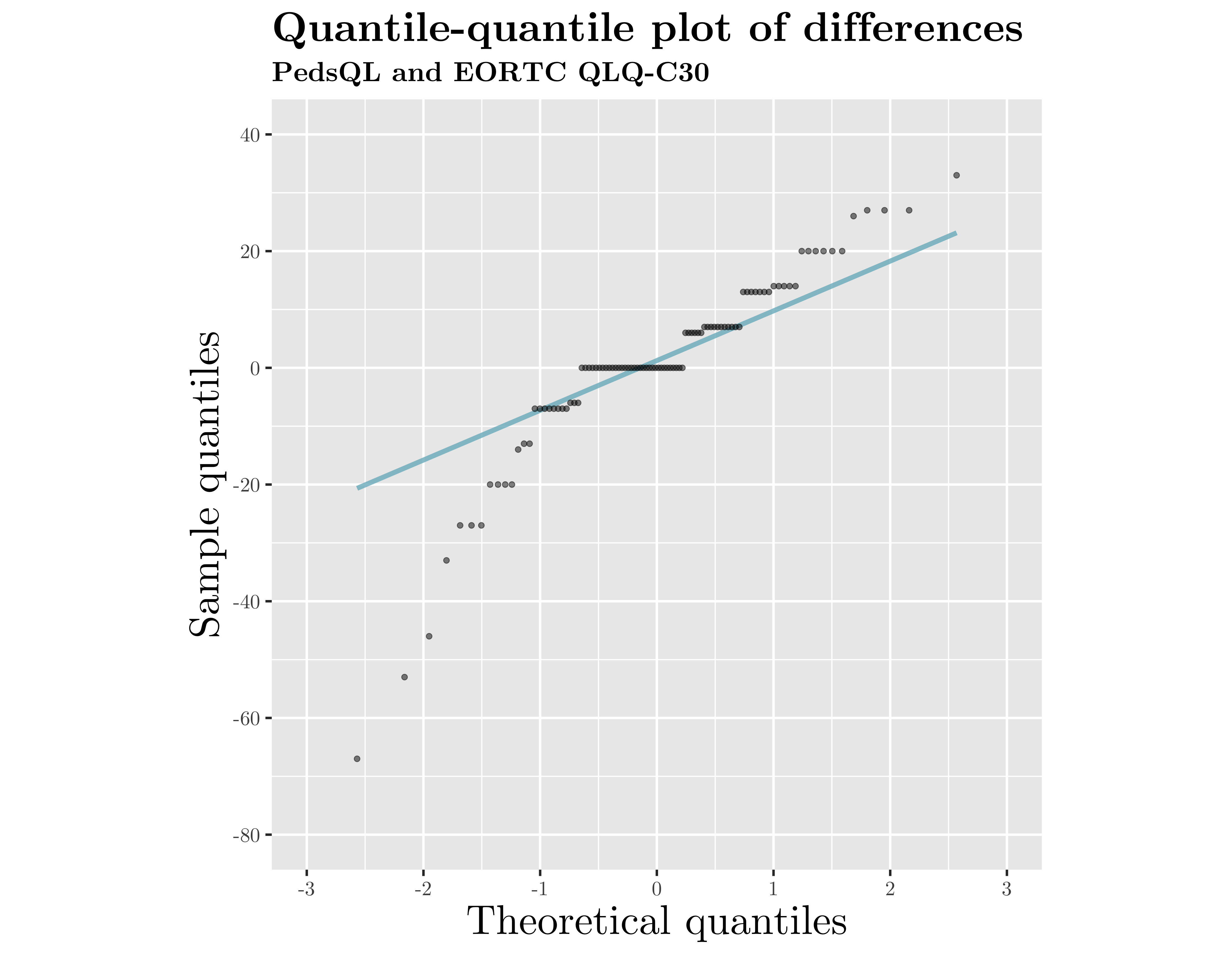

Supplement: Multimedia component 1 [file mmc1.zip › pedsql_eortc_qq_plot.png]

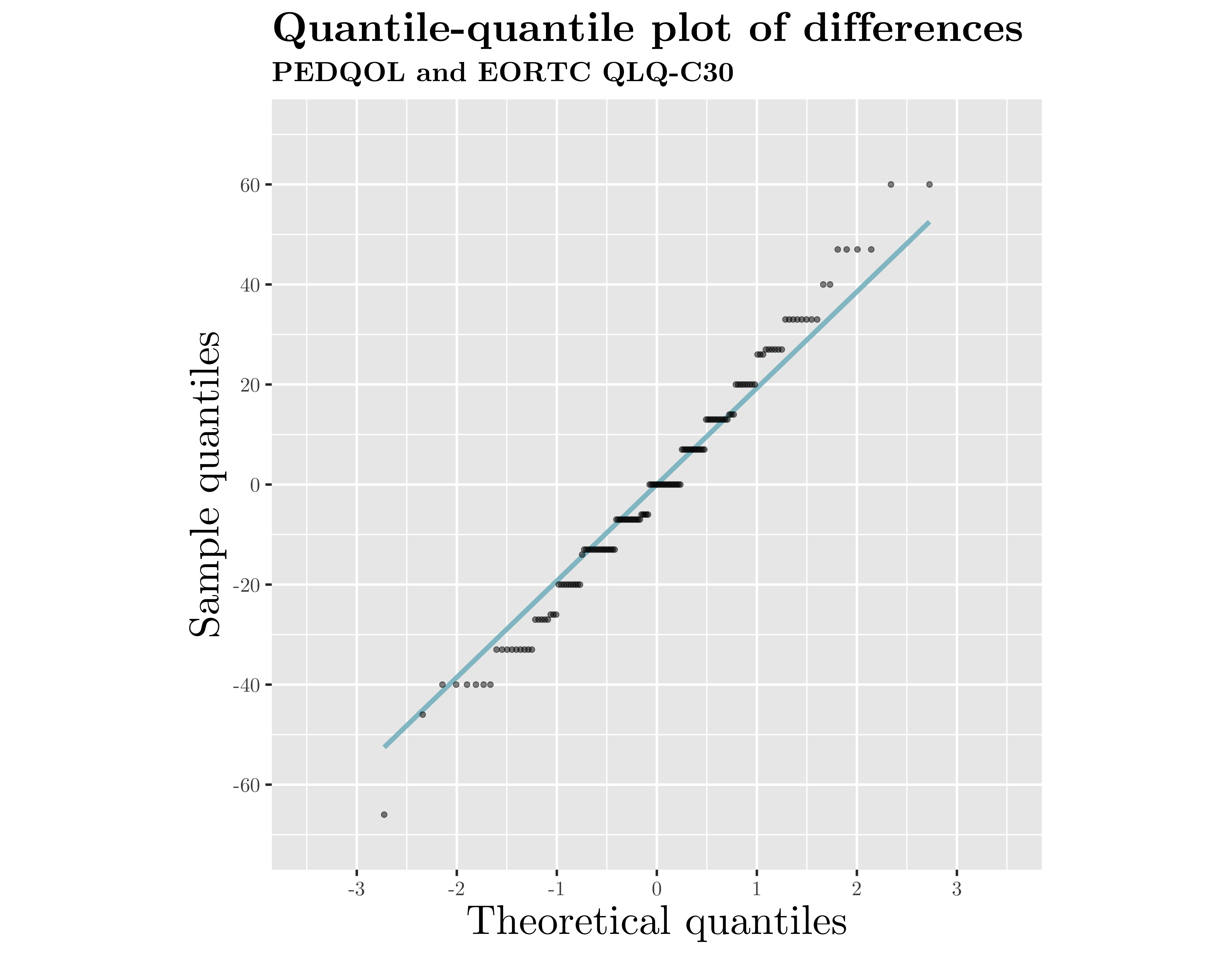

Supplement: Multimedia component 1 [file mmc1.zip › pedqol_eortc_qq_plot.png]

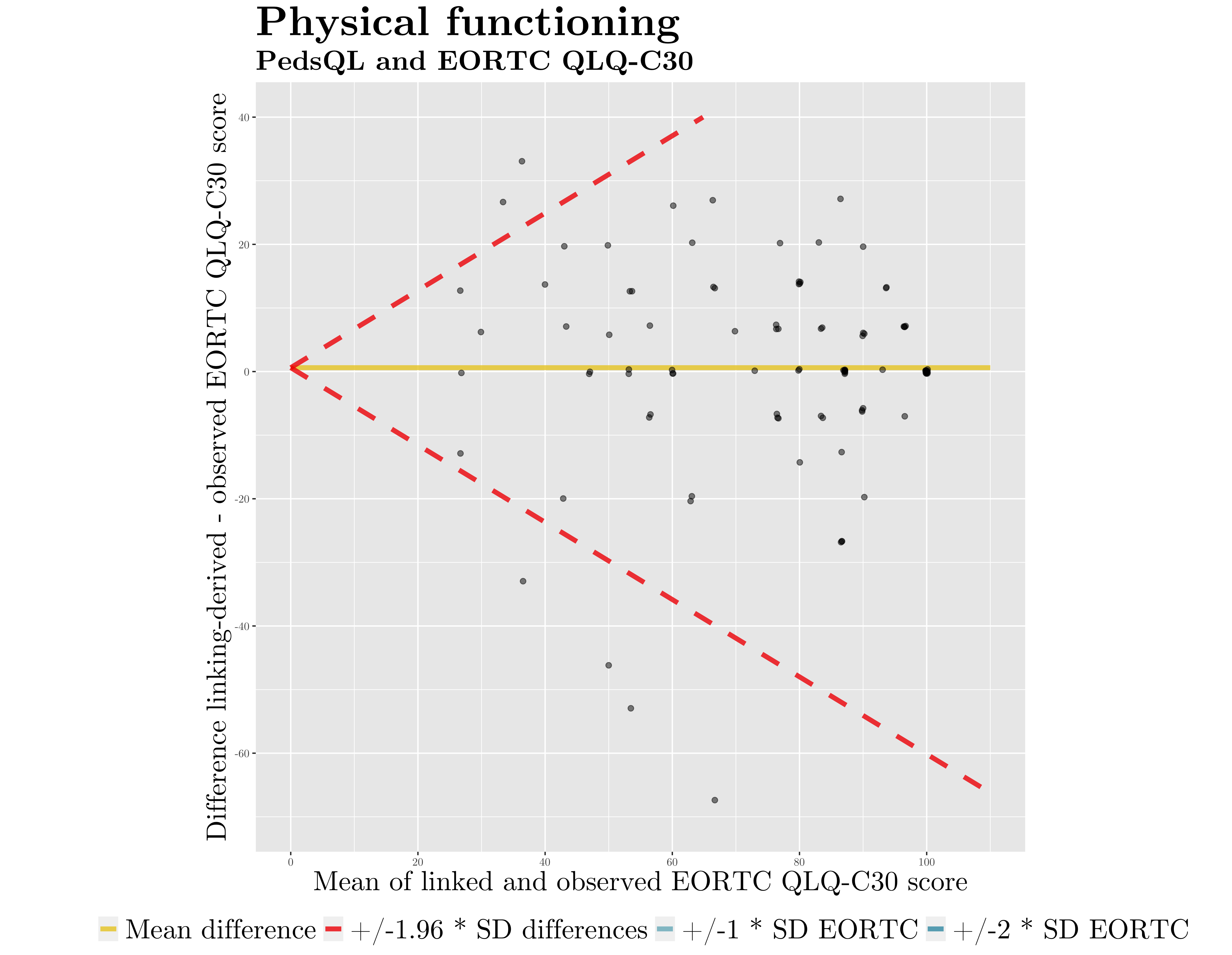

Supplement: Multimedia component 1 [file mmc1.zip › pedsql_eortc_physical_bland_altman_log_back.png]

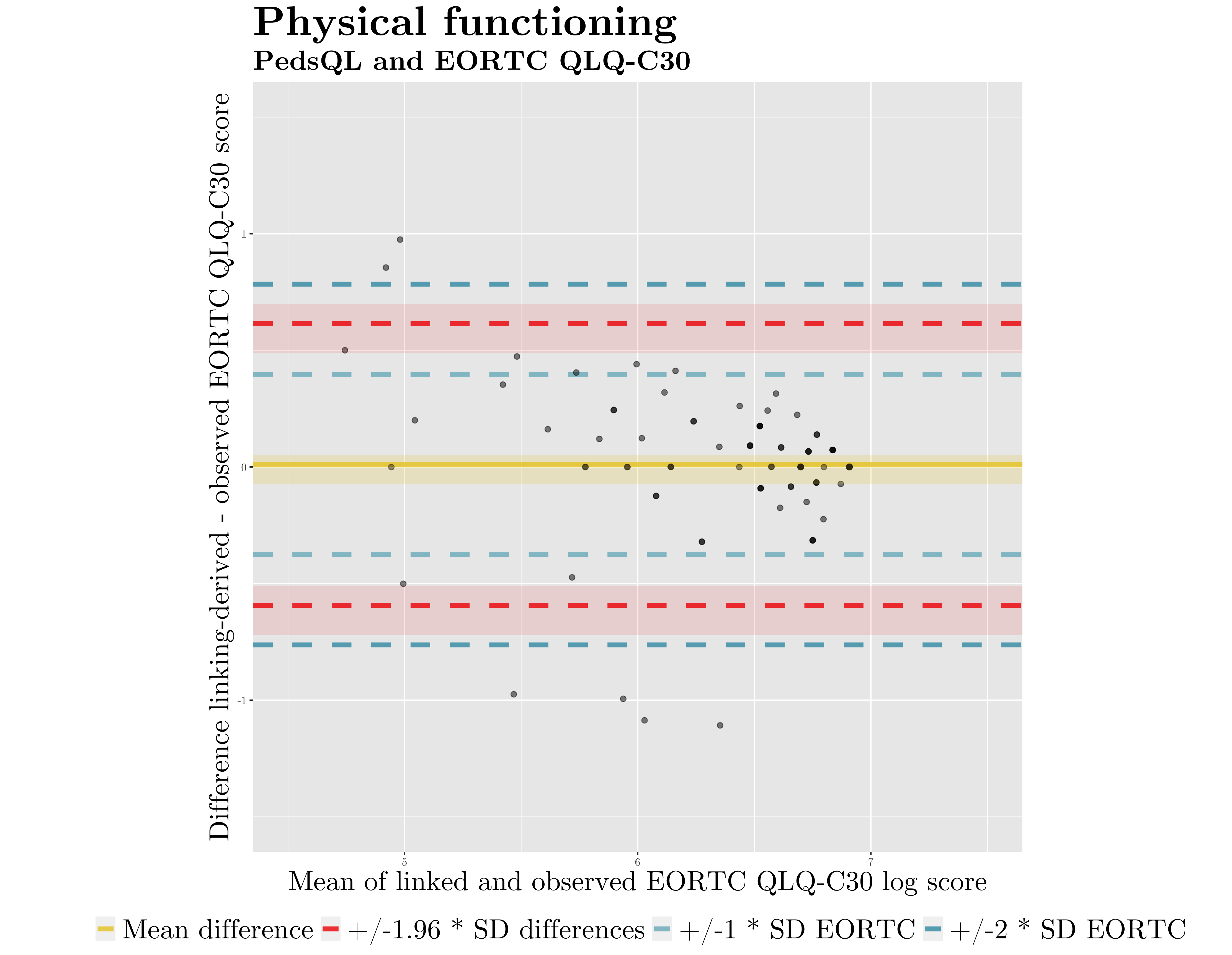

Supplement: Multimedia component 1 [file mmc1.zip › pedsql_eortc_physical_bland_altman_log.png]

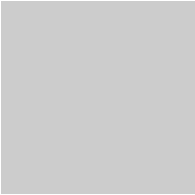

Supplement: Multimedia component 1 [file mmc1.zip › figs/Fig3.pdf]

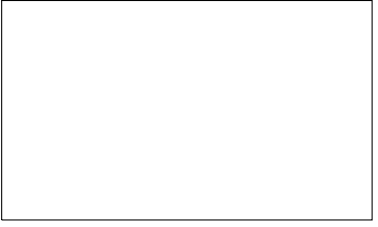

Supplement: Multimedia component 1 [file mmc1.zip › figs/grabs.pdf]

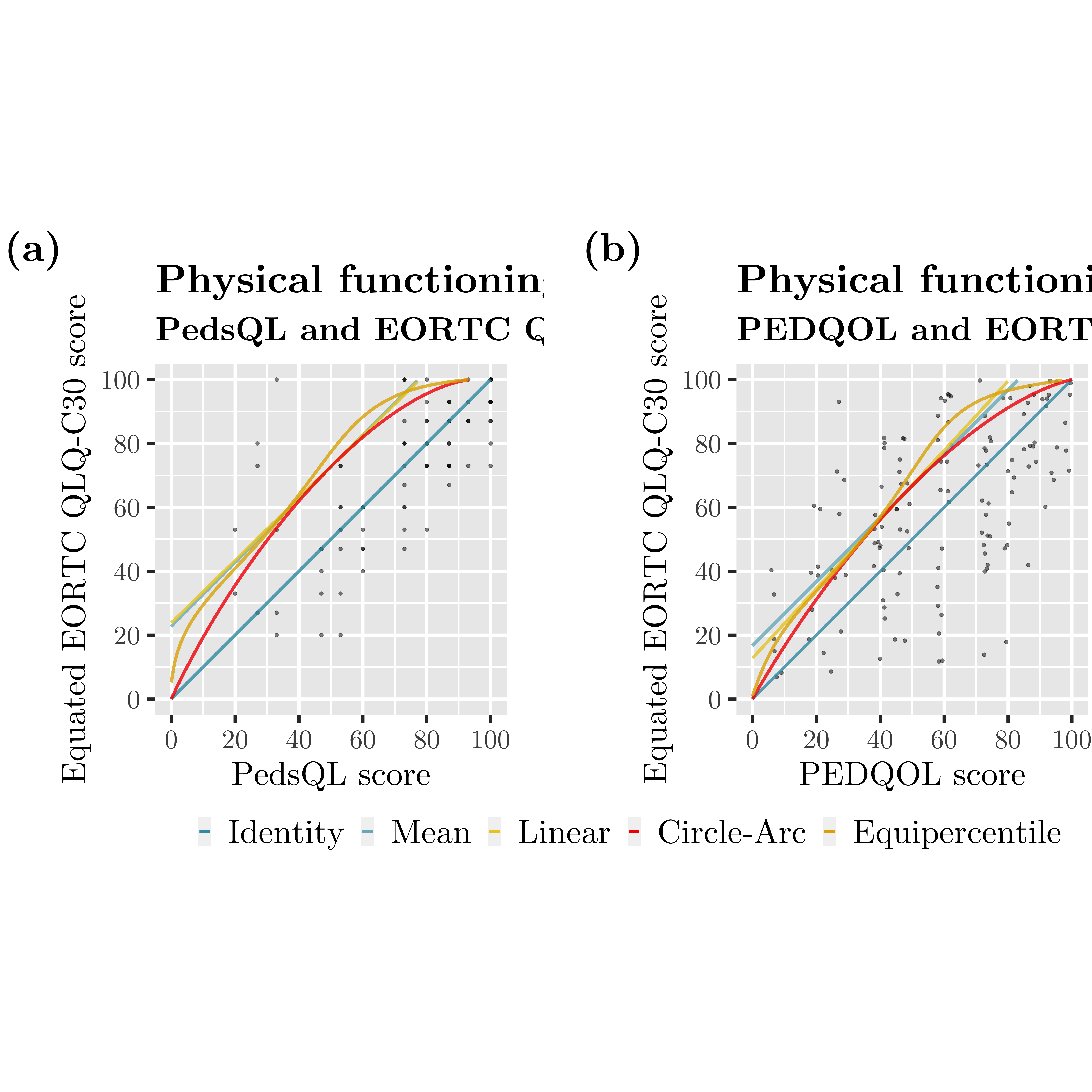

Supplement: Multimedia component 1 [file mmc1.zip › figs/functions-physical.png]

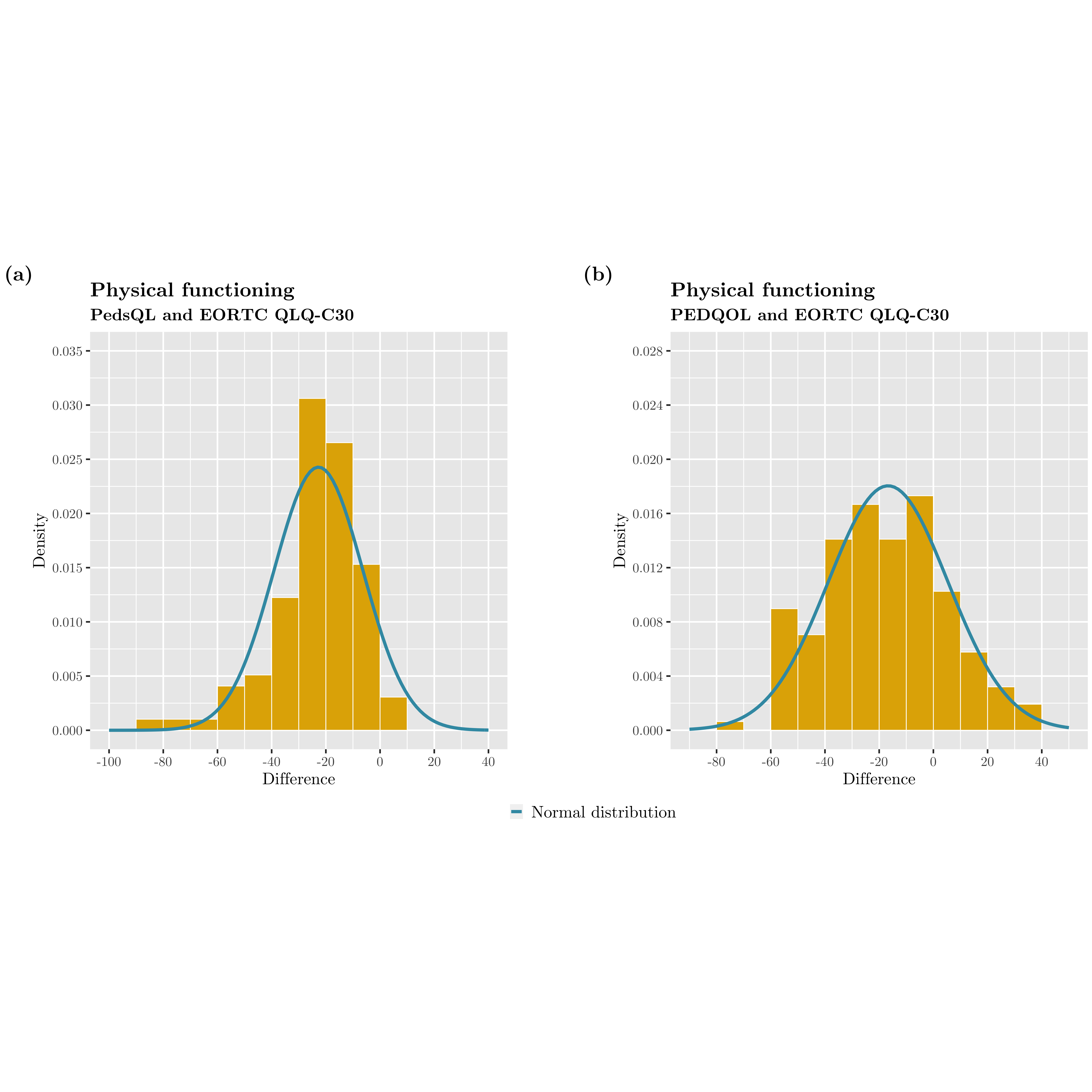

Supplement: Multimedia component 1 [file mmc1.zip › figs/physical-histogram.png]

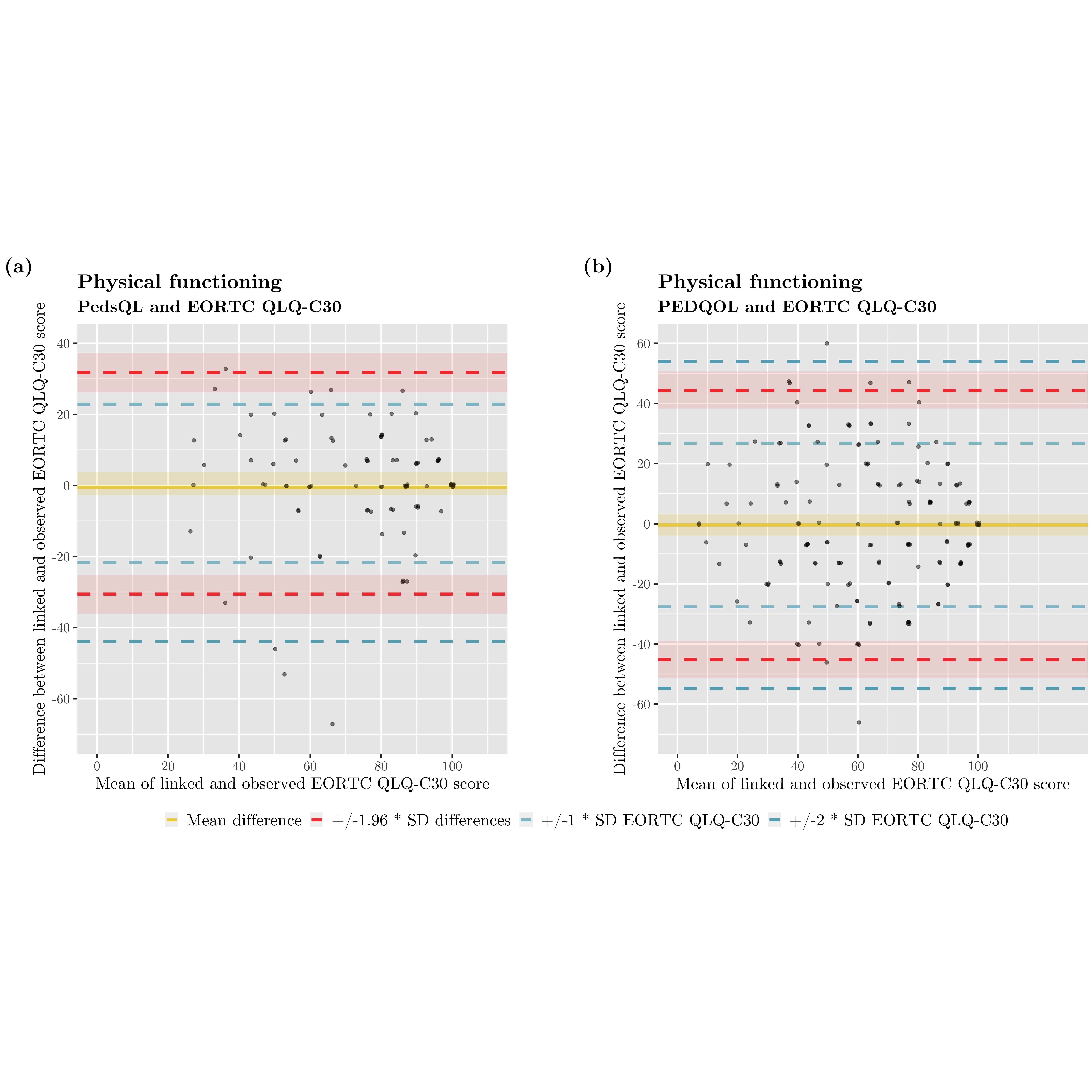

Supplement: Multimedia component 1 [file mmc1.zip › figs/bland-altman-physical.png]

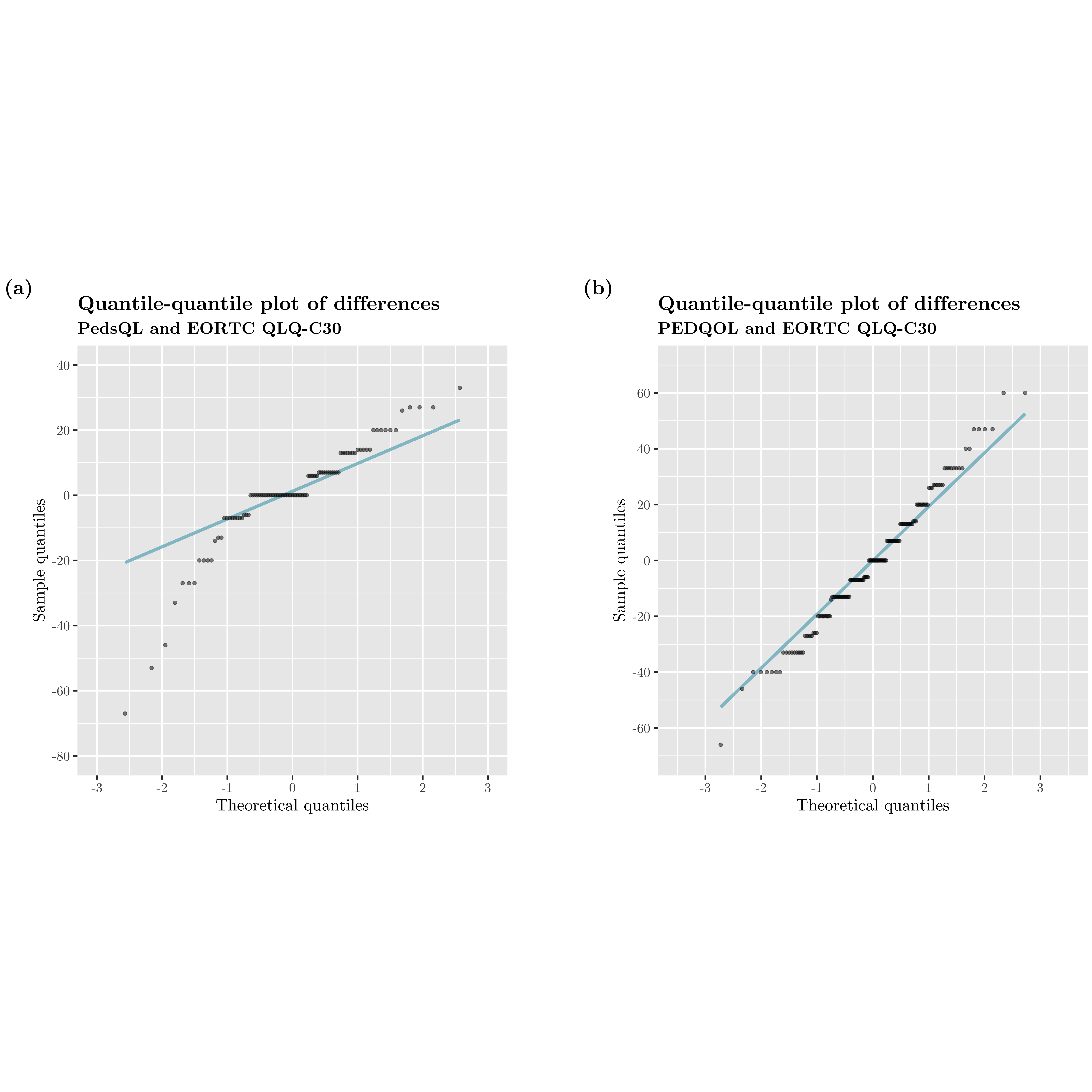

Supplement: Multimedia component 1 [file mmc1.zip › figs/physical-qqplot.png]

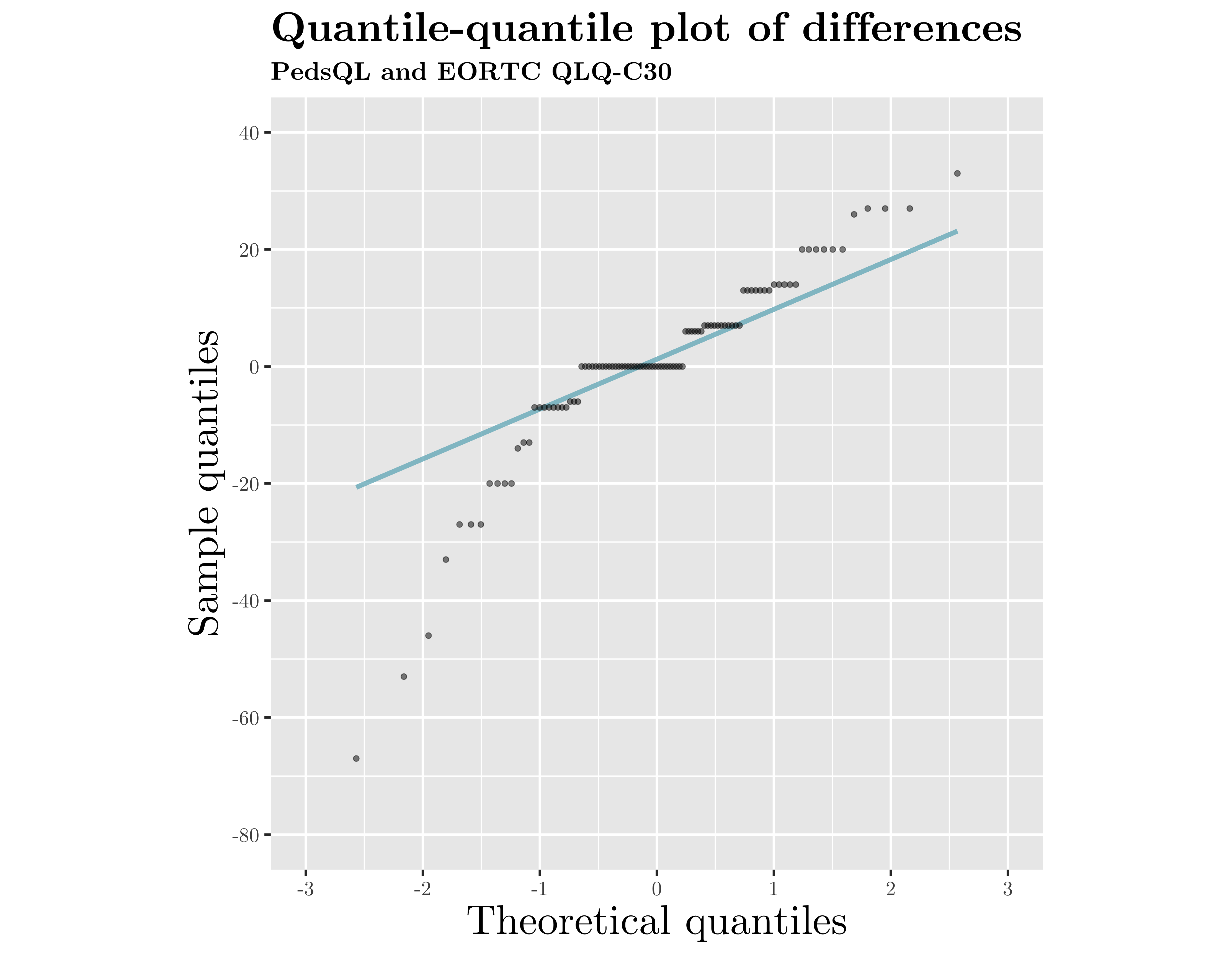

Supplement: Multimedia component 1 [file mmc1.zip › figs/pedsql_eortc_qq_plot.png]

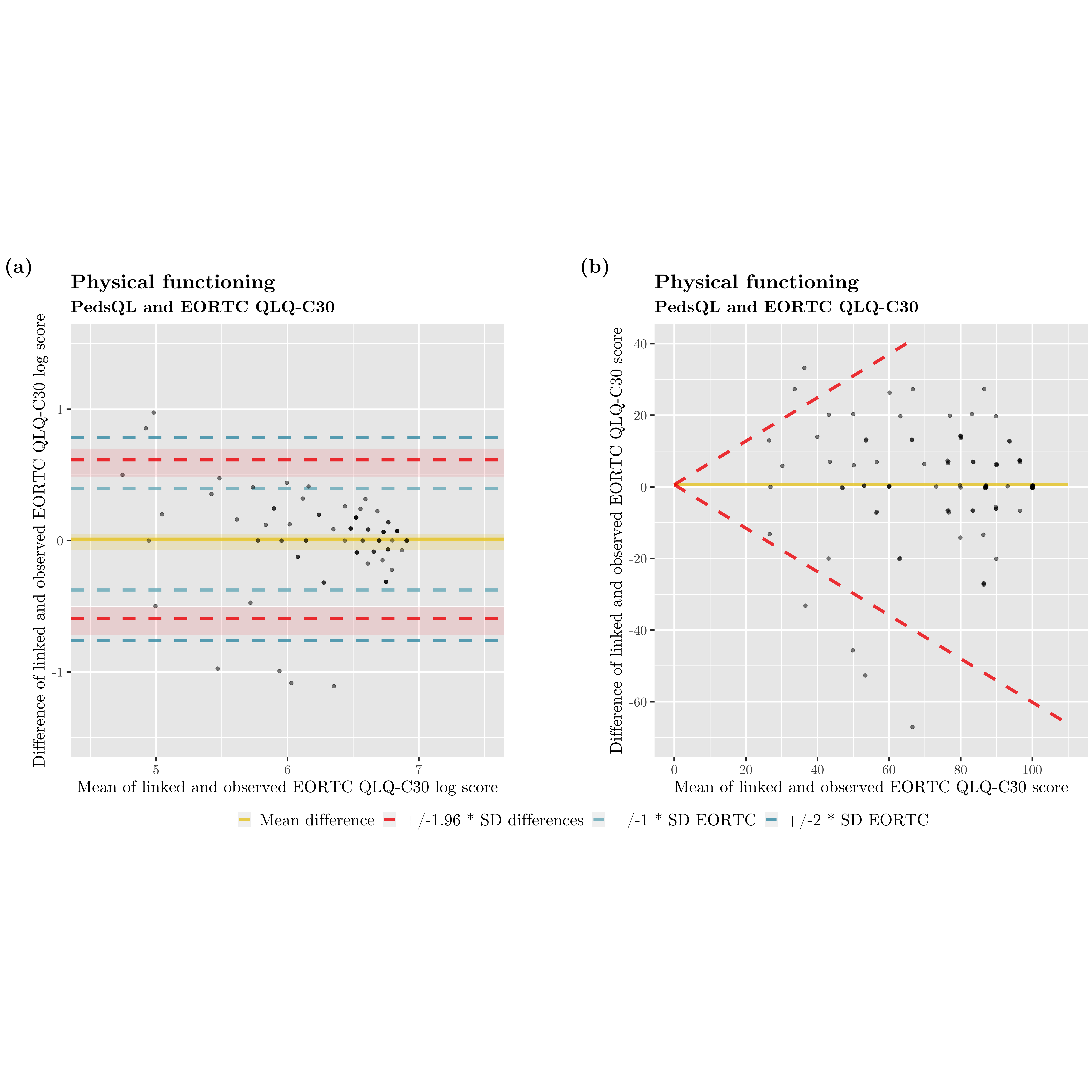

Supplement: Multimedia component 1 [file mmc1.zip › figs/bland-altman-physical-log.png]

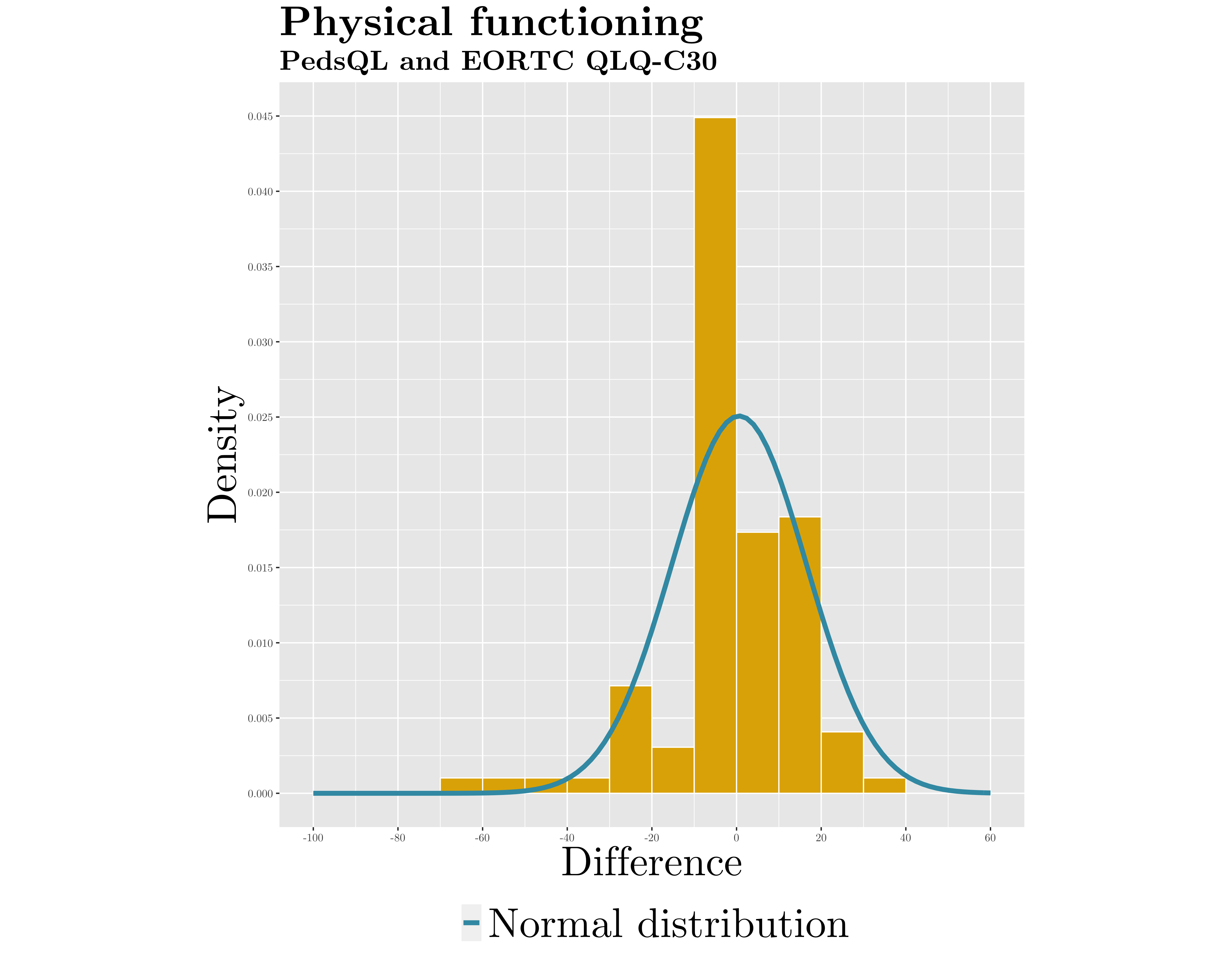

Supplement: Multimedia component 1 [file mmc1.zip › figs/pedsql_eortc_physical_histogram.png]

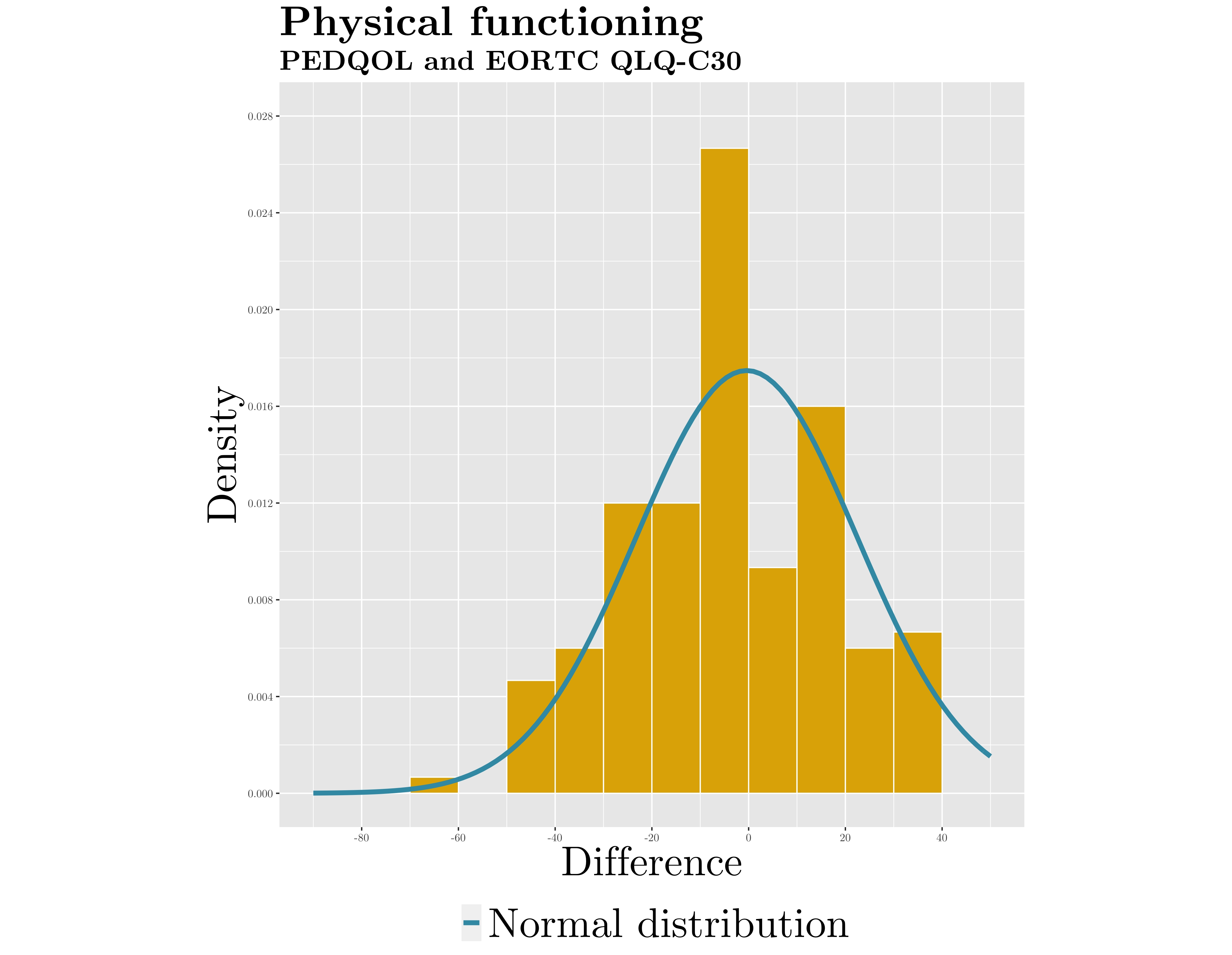

Supplement: Multimedia component 1 [file mmc1.zip › figs/pedqol_eortc_physical_histogram.png]

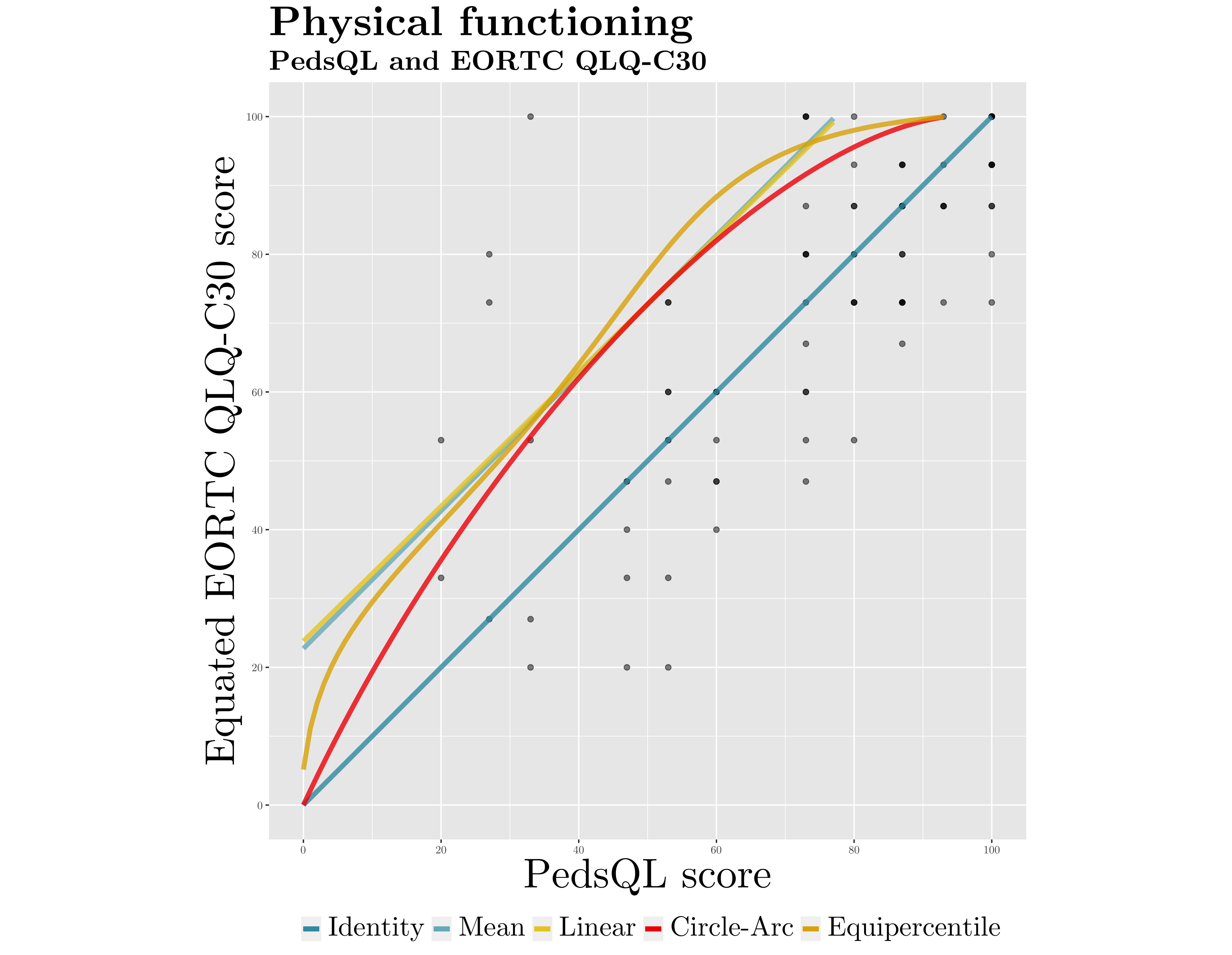

Supplement: Multimedia component 1 [file mmc1.zip › figs/pedsql_eortc_functions_physical.png]

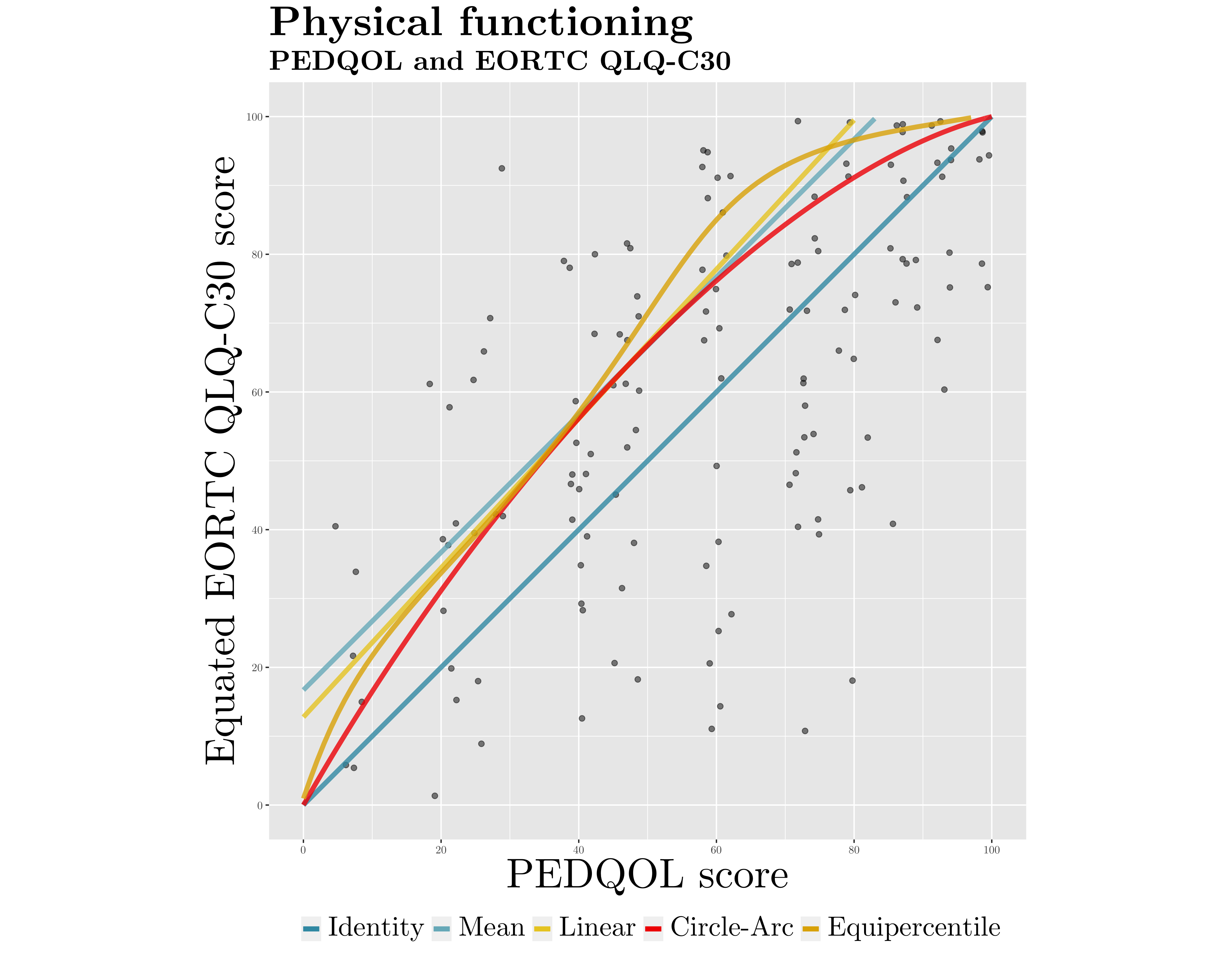

Supplement: Multimedia component 1 [file mmc1.zip › figs/pedqol_eortc_functions_physical.png]

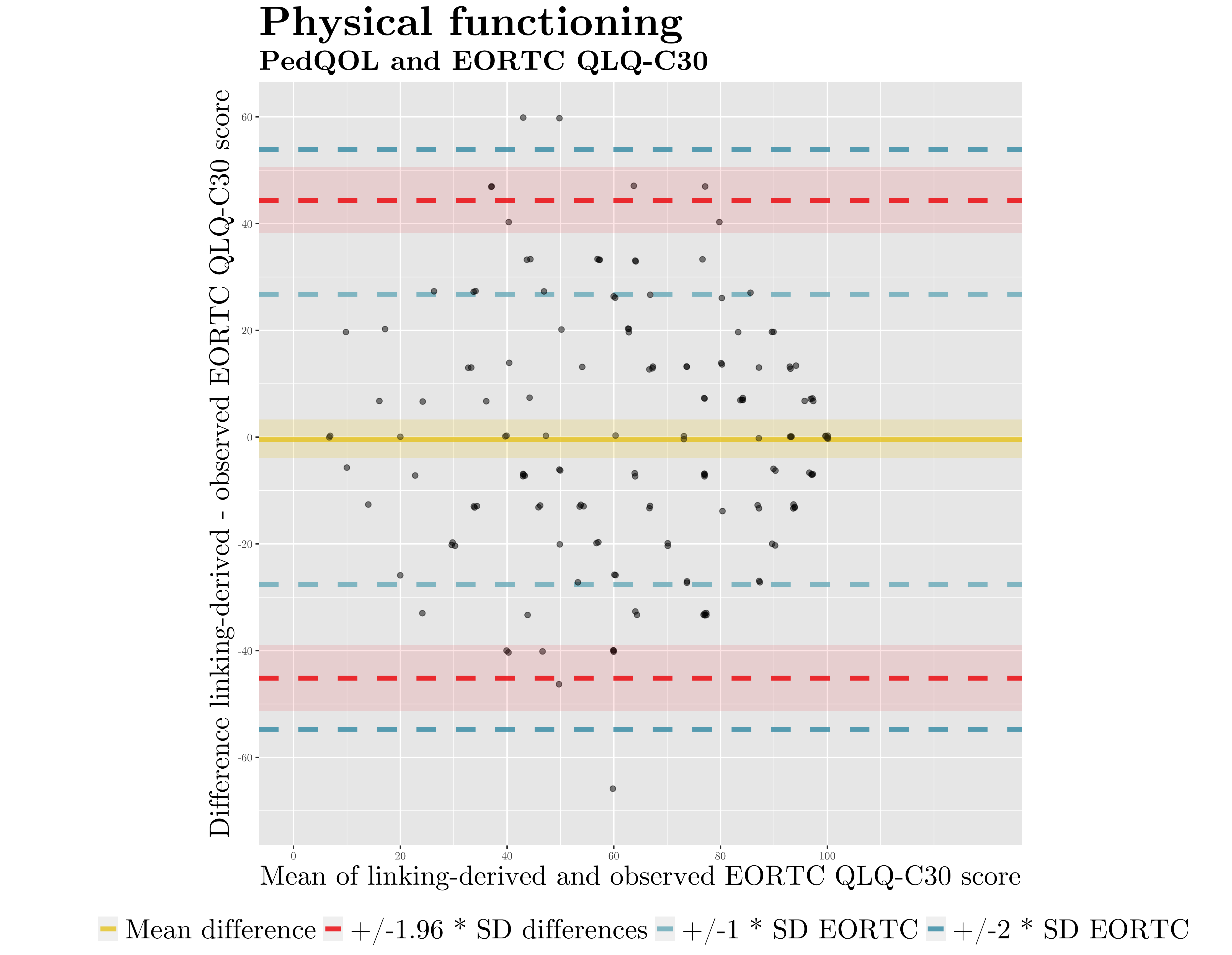

Supplement: Multimedia component 1 [file mmc1.zip › figs/pedqol_eortc_physical_bland_altman.png]

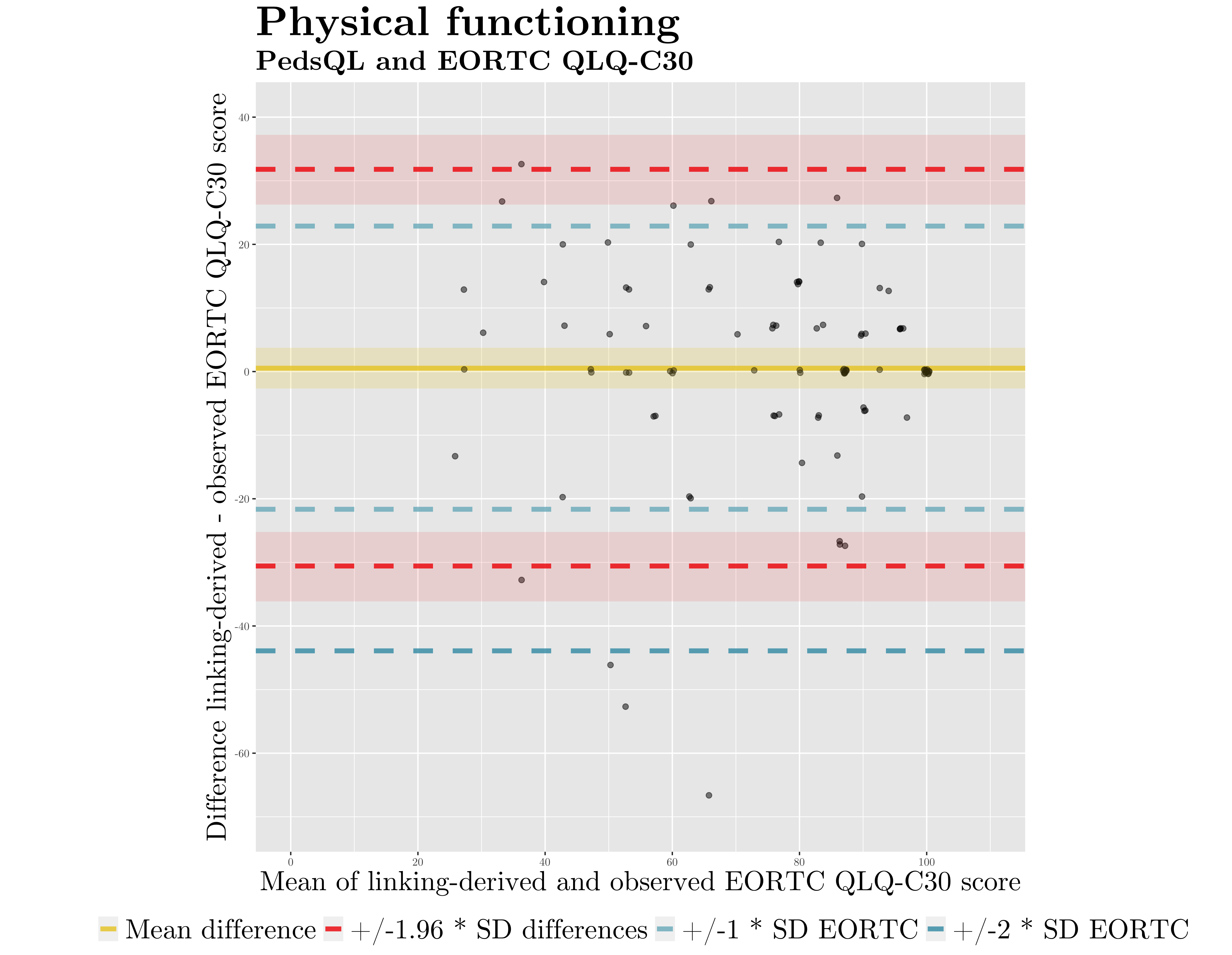

Supplement: Multimedia component 1 [file mmc1.zip › figs/pedsql_eortc_physical_bland_altman.png]

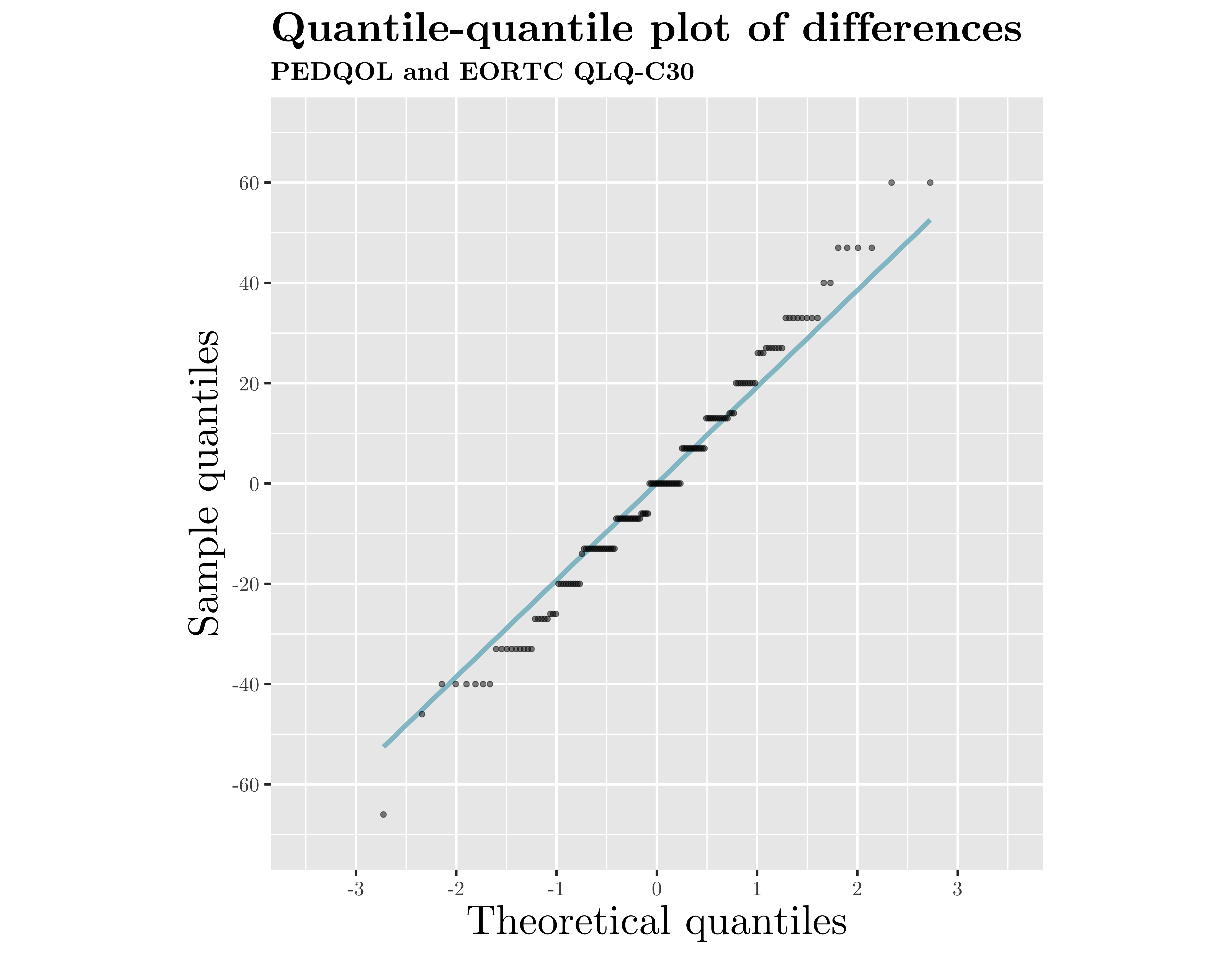

Supplement: Multimedia component 1 [file mmc1.zip › figs/pedqol_eortc_qq_plot.png]

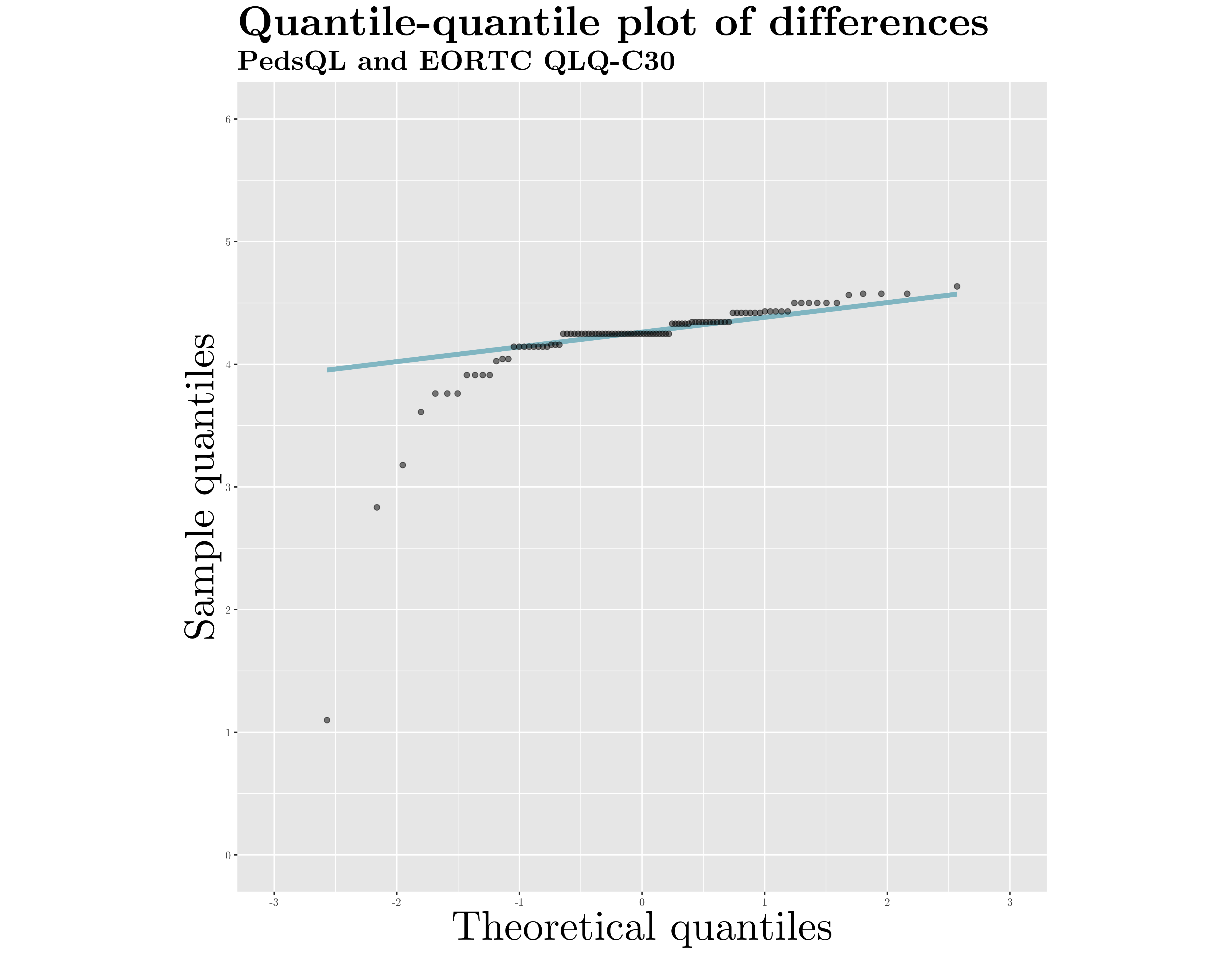

Supplement: Multimedia component 1 [file mmc1.zip › figs/pedsql_eortc_qq_log_plot.png]

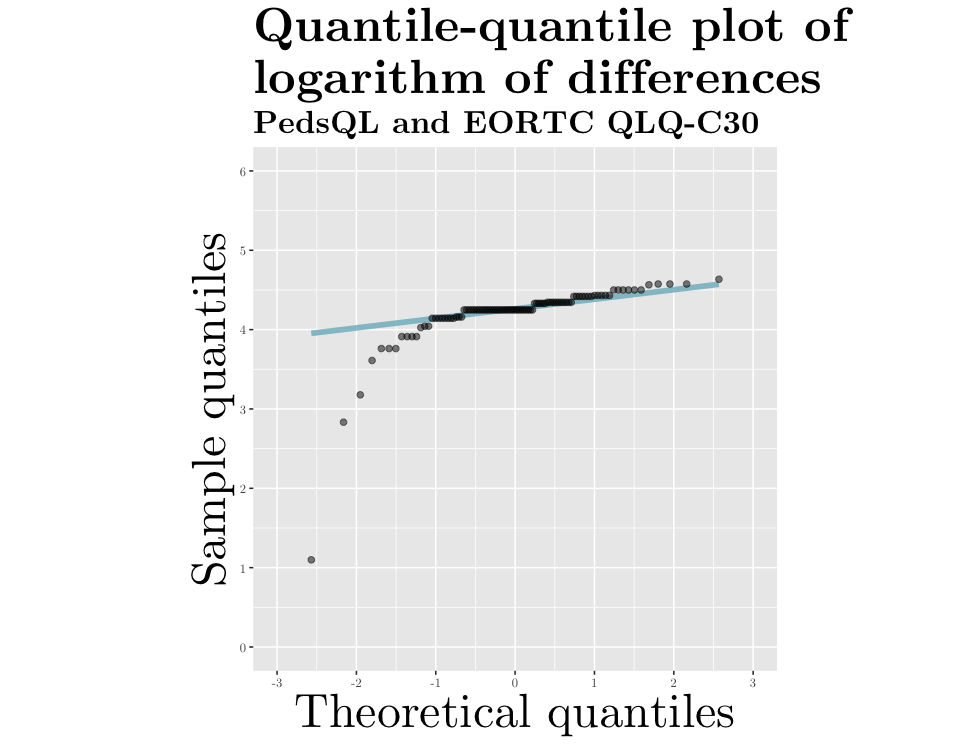

Supplement: Multimedia component 1 [file mmc1.zip › figs/Fig. 6.png]

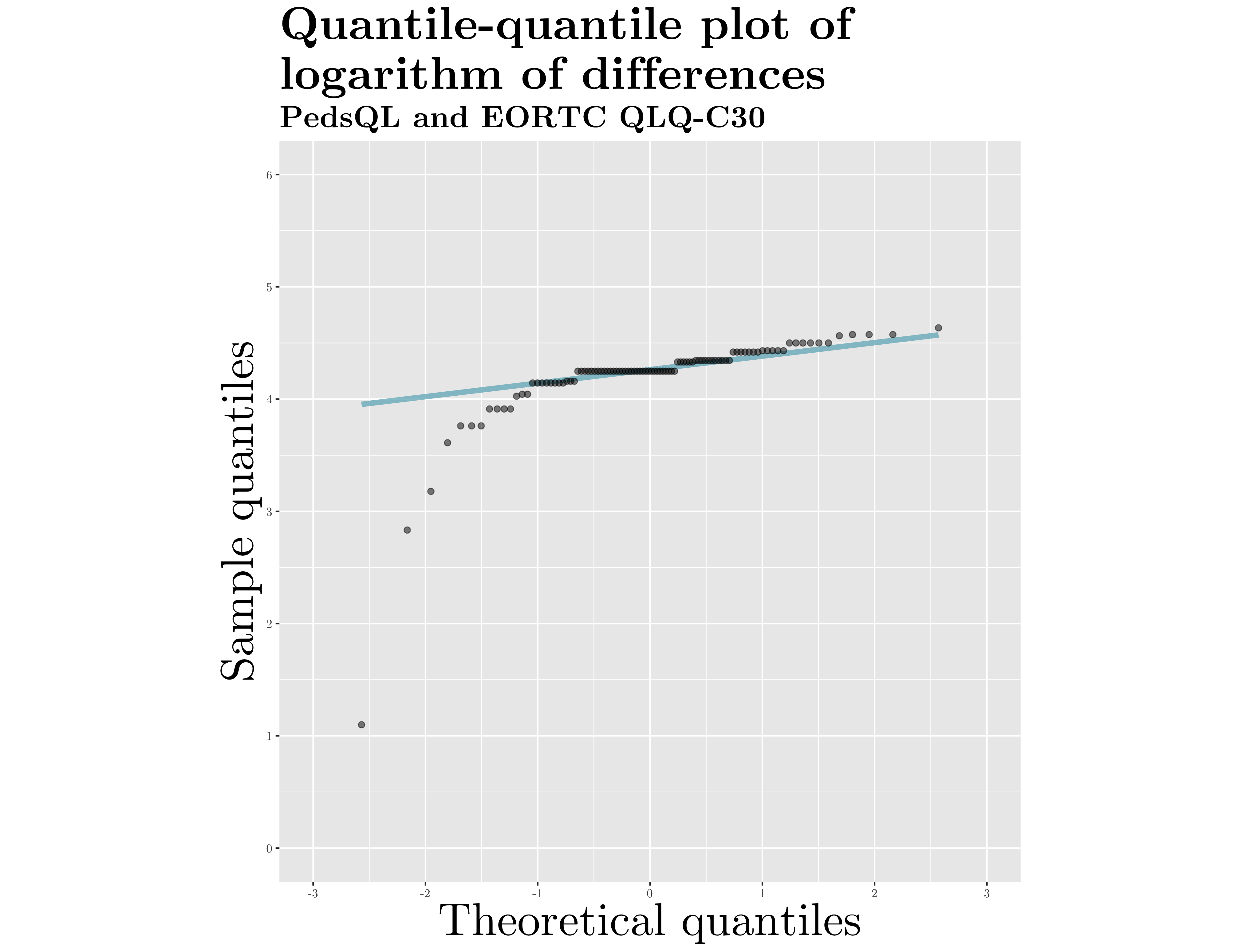

Supplement: Multimedia component 1 [file mmc1.zip › figs/test.png]

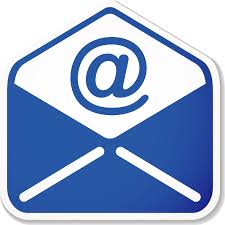

Supplement: Multimedia component 1 [file mmc1.zip › thumbnails/cas-email.jpeg]

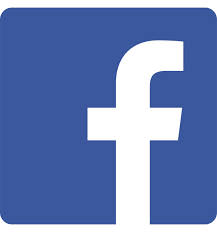

Supplement: Multimedia component 1 [file mmc1.zip › thumbnails/cas-facebook.jpeg]

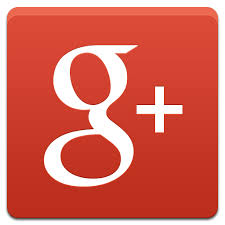

Supplement: Multimedia component 1 [file mmc1.zip › thumbnails/cas-gplus.jpeg]

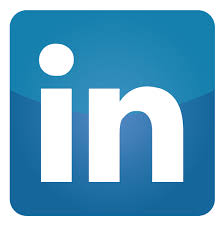

Supplement: Multimedia component 1 [file mmc1.zip › thumbnails/cas-linkedin.jpeg]

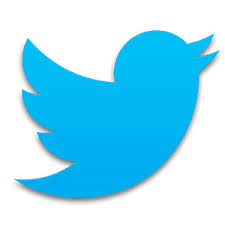

Supplement: Multimedia component 1 [file mmc1.zip › thumbnails/cas-twitter.jpeg]

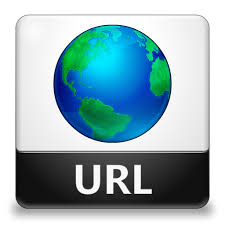

Supplement: Multimedia component 1 [file mmc1.zip › thumbnails/cas-url.jpeg]
